# Developmental Brain Network Trajectories Differentiate Resilience and Vulnerability to Psychosis in 22q11.2 Deletion Syndrome

**DOI:** 10.1016/j.bpsgos.2026.100768

**Published:** 2026-06-10

**Authors:** Silas Forrer, Farnaz Delavari, Corrado Sandini, Dimitri Van De Ville, Stephan Eliez

**Affiliations:** a Developmental Imaging and Psychopathology Laboratory, University of Geneva School of Medicine, Geneva, Switzerland; b Medical Image Processing Laboratory, Neuro-X Institute, École Polytechnique Fédérale de Lausanne, Geneva, Switzerland; c Department of Radiology and Medical Informatics, University of Geneva, Geneva, Switzerland; d Center for Biomedical Imaging, Geneva, Switzerland; e Department of Genetic Medicine and Development, University of Geneva School of Medicine, Geneva, Switzerland

**Keywords:** 22q11 deletion syndrome, Connectome fingerprinting, Longitudinal imaging, Network stability, Psychosis risk, Resting-state fMRI

## Abstract

**Background:**

Disrupted maturation of functional brain networks has been increasingly linked to elevated risk of psychosis, but the spatiotemporal characteristics of these deviations remain unclear. We used longitudinal connectome fingerprinting and jointly analyzed the functional connectivity (FC) and intraclass correlation coefficient (ICC) to obtain age-dependent trajectories of functional brain organization in 22q11.2 deletion syndrome (22q11DS), a high-risk model for psychosis.

**Methods:**

Resting-state functional magnetic resonance imaging from 62 individuals with 22q11DS and 63 control participants (ages 8–30 years, 2–5 visits) were analyzed. Patients were stratified by the presence or absence of positive psychotic symptoms (PPSs) [PPS(+), PPS(−)]. The FC and ICC characterization of connectome fingerprints were projected onto a joint principal component (PC) space, yielding 3 orthogonal axes of maturation: PC1 (sensory-association axis), PC2 (emotional-cognitive balance), and PC3 (executive-sensory control). Longitudinal trajectories along these axes characterized the directionality and coordination of connectome maturation.

**Results:**

Control participants showed a clear decrease in identifiability and stability during adolescence, followed by recovery into adulthood, reflecting coordinated, direct maturation along all 3 axes. PPS(−) individuals exhibited a partial decrease and subsequent recovery, preserving adaptive reorganization along PC3. In contrast, PPS(+) participants displayed disorganized and irregular trajectories in the PC space and a decline in PC3, indicating disrupted temporal coordination of network maturation and fragmented executive-sensory integration.

**Conclusions:**

Loss of developmental synchrony in FC was associated with psychosis vulnerability in 22q11DS. PC analysis–based multiaxis mapping revealed that resilience and vulnerability are determined by the direction and synchrony of brain maturation rather than by stability alone, highlighting a novel marker for tracking neurodevelopmental risk across psychiatric disorders.

## Plain Language Summary

People with 22q11.2 deletion syndrome are at higher risk for developing psychosis, but how functional brain networks mature in those who do versus do not develop symptoms remains unclear. In this study, we followed children, adolescents, and young adults with 22q11.2 deletion syndrome and healthy peers, using brain imaging to track changes in functional connectivity over time. We found that individuals without psychotic symptoms showed typical adolescent reorganization of brain networks, while those with early psychotic symptoms displayed irregular and less coordinated network development. These findings reveal brain maturation patterns that may protect against or contribute to psychosis.

The dysconnectivity hypothesis of schizophrenia, which proposes that psychotic symptoms arise from disrupted communication between brain regions, remains a cornerstone of psychiatric neuroscience (1,2). Despite substantial evidence from neuroimaging and neurophysiological studies supporting abnormal functional integration in schizophrenia, the concept remains mechanistically underspecified, and clinically useful biomarkers remain limited. Existing candidate biomarkers, including structural and functional imaging measures (3, 4, 5, 6, 7), electroencephalography- or magnetoencephalography-based measures of altered connectivity or oscillatory coupling (8, 9, 10, 11, 12, 13), and molecular markers related to synaptic plasticity (2,14, 15, 16), have yet to achieve sufficient specificity or robustness for routine clinical application. Brain connectivity can be examined at multiple levels: structural connectivity, reflecting the anatomical white matter pathways linking regions, and functional connectivity (FC), the temporal coordination of neural activity between regions. While structural connectivity provides the physical scaffold for information transfer, FC captures dynamic patterns of coordinated activity. However, it remains unclear whether dysconnectivity primarily reflects overly strong or weak coupling, excessive temporal instability, or a breakdown of large-scale network coordination and whether these alterations remain stable or fluctuate across development.

One promising approach to answering these open questions is connectome fingerprinting, which leverages the unique and stable patterns of FC within individuals (17). Fingerprinting methods have demonstrated that individual functional connectomes are sufficiently distinct to identify individuals with high accuracy, reflecting both functional network stability and individual specificity (18, 19, 20). In healthy populations, connectome fingerprinting has largely focused on test-retest designs over short time scales (typically days or weeks), showing a favorable trade-off between fingerprint stability corresponding to greater within-individual consistency and distinctiveness reflecting unique connectivity patterns compared with others (21,22). Emerging work has begun to apply fingerprinting to clinical populations, revealing alterations in network stability and identifiability in conditions such as cognitive decline, Alzheimer’s disease, autism spectrum disorder, attention-deficit/hyperactivity disorder, and mood disorders (23, 24, 25, 26). However, these studies have predominantly used adult samples and short test-retest intervals, leaving open the question of how connectome stability and individualization unfold across neurodevelopment.

This gap is particularly relevant for psychosis, increasingly understood as a neurodevelopmental disorder characterized by atypical maturation of brain structure and function (27,28). Longitudinal studies with youths at elevated risk for psychosis have revealed altered cortical thinning, disrupted network segregation, and atypical developmental trajectories of connectivity during adolescence (29, 30, 31, 32). However, studying these mechanisms in the general population is challenging due to the low incidence of psychosis, substantial phenotypic heterogeneity, and the difficulty of identifying individuals early enough to capture key maturational windows. These limitations underscore the need for developmental approaches applied within populations in which risk for psychosis is both elevated and identifiable early in life.

Genetic high-risk models offer a powerful approach to overcoming these limitations. 22q11.2 deletion syndrome (22q11DS), in particular, provides a unique neurodevelopmental model, with approximately 30% of individuals developing psychotic disorders, a rate 20 to 30 times higher than the general population (33,34). This elevated and predictable risk enables prospective longitudinal investigation of network maturation during the critical developmental period when vulnerability to psychosis emerges. Despite this opportunity, no studies have applied longitudinal connectome fingerprinting to examine how FC stability and distinctiveness evolve across development in high-risk clinical populations. While fingerprinting has been applied across the lifespan and multiple visits, they still use mainly cross-sectional designs (e.g., test-retest) and thus have not captured the dynamic maturational changes occurring across critical periods (35, 36, 37).

To address this gap, we developed a longitudinally adapted connectome fingerprinting framework (24) designed to characterize developmental changes in functional synchrony and within-individual FC stability. Using a moving-window approach, we computed identification rate (IdRate) and intraclass correlation coefficients (ICCs) across overlapping age periods, enabling us to track the unfolding of connectome maturation from late childhood to adulthood. In addition, we applied principal component analysis (PCA) to the combined functional connectomes and ICC matrices across windows, yielding a low-dimensional representation of developmental trajectories.

In a longitudinal context with multiyear follow-ups, high connectome similarity for one individual across years, especially across adolescence, which represents a period of profound brain plasticity, may not simply reflect healthy stability. Rather, it could indicate prematurely stabilized or inflexible networks. Inversely, temporary dips in identifiability may reflect adaptive reorganization needed for maturation. Thus, in this developmental framework, IdRate and ICC should be interpreted not as static measures of network rigidity but as developmental indices revealing whether functional networks are undergoing timely reorganization appropriate to each developmental stage. This reframing is particularly crucial when examining individuals with 22q11DS, as divergence in connectome patterns may signal either inadequate neural reorganization needed for maturation or, conversely, adaptive flexibility that supports resilience.

Based on prior work on fingerprinting and network dynamics in psychosis-related conditions (31,38, 39, 40, 41), we hypothesized that the typical developmental trajectory, characterized by a transient decrease in connectome identifiability during adolescence due to network reorganization followed by increasing stability and individualization in adulthood, would be altered in 22q11DS (42,43). Specifically, given the elevated risk for psychosis in this population and prior reports of altered network development and reduced connectome distinctiveness in schizophrenia and ultra-high-risk groups (44,45), we expected individuals with 22q11DS to show atypical maturation trajectories in the PC space, varying as a function of psychotic symptom emergence.

## Methods and Materials

### Participants

The dataset comprised imaging and behavioral data from 125 participants, including 62 participants with a genetically confirmed diagnosis of 22q11DS (28 females, age range = 8.1–34.0 years) and 63 healthy control (HC) participants (30 females, age range = 7.4–33.2 years). The number of longitudinal assessments varied between 2 and 5, resulting in 307 longitudinal assessments overall (160 for 22q11.2DS carriers and 147 for HC participants). Demographic characteristics, medication status, and psychiatric comorbidities were systematically assessed and are summarized in Table 1. Written informed consent was obtained from participants and/or their parents. The study was approved by the cantonal ethics committee and conducted in accordance with the Declaration of Helsinki.

**Table 1:** Participant Characteristics

Demographic Variables	22q11DS	HC	*p* Value
Number of Participants, Female/Male	62, 31/31	63, 34/29	.66∗
Number of Scans, Female/Male	160, 83/77	147, 79/68	.44∗
Number of Participants Having 1 Scan	0	0	NA
Number of Participants Having 2 Scans	37	42	NA
Number of Participants Having 3 Scans	16	21	NA
Number of Participants Having 4 Scans	7	0	NA
Number of Participants Having 5 Scans	2	0	NA
Average Time Between Longitudinal Visits, Years	4.01 (1.33)	3.88 (1.04)	.44^a^
PPS(+) Participants, Female/Male	40, 22/18	0	.34∗
PPS(−) Participants, Female/Male	17, 7/10	0	
Scanner Type: Prisma-Fit/Trio	51/109	36/111	.15
Average Age, Years	19.60 (5.75)	17.20 (6.26)	<.001∗∗
Average IQ, Years	71.41 (11.35)	113.25 (12.56)	<.001∗∗
Average FD After Scrubbing	0.16 (0.04)	0.13 (0.04)	<.001∗∗
Anxiety Disorder^b^	34 (54.8%)	0	NA
Attention-Deficit/Hyperactivity Disorder^b^	34 (54.8%)	0	NA
Mood Disorder^b^	19 (30.6%)	0	NA
Schizophrenia Spectrum Disorders^b^	8 (12.9%)	0	NA
More Than 1 Psychiatric Comorbidity^b^	31 (62.0%)	0	NA
Anticonvulsants^b^	3 (4.8%)	0	NA
Antidepressants^b^	25 (40.2%)	0	NA
Neuroleptic^b^	14 (22.6%)	0	NA
Psychostimulant^b^	27 (43.5%)	0	NA
Anxiolytic^b^	3 (4.8%)	0	NA
Antiepileptic^b^	2 (3.2%)	0	NA

Values are presented as *n*, mean (SD), or *n* (%). IQ was measured using the Wechsler Intelligence Scale for Children-III (76) for children and the Wechsler Adult Intelligence Scale-III (77) for adults. Presence of psychiatric disorders was determined using clinical interview with patients using the Diagnostic Interview for Children and Adolescents-Revised (78), the psychosis supplement from the Schedule for Affective Disorders and Schizophrenia for School-Age Children–Present and Lifetime version (79), and the Structured Clinical Interview for DSM-IV Axis I disorders (80).

Significant at the level of ∗*p* < .05 (χ^2^ test), ∗∗*p* < .05 (*t* test).

22q11DS, 22q11 deletion syndrome; FD, framewise displacement; HC, healthy control; NA, not available; PPS, positive psychotic symptoms; PPS(+), with PPS; PPS(−), without PPS.

a Calculated first-level average within-subject measurements and then second-level across all participants.

b Positive if present at any of the repetitive assessments.

### Clinical Assessment

To investigate the implications of individual developmental deviation in the proneness to psychosis, we created subgroups with positive psychotic symptoms [PPS(+)] and without PPS [PPS(−)] using the Structured Interview for Prodromal Symptoms, an adapted and validated instrument in 22q11.2DS (46, 47). This clinical tool was used to assess the presence and the severity of PPSs at each time point. The positive subscale includes the 5 following symptoms: unusual thought content (P1), suspiciousness (P2), grandiose ideas (P3), hallucinations (P4), and disorganized communication (P5). Five participants were excluded due to too young age for clinical assessment or missing data points. For the rest of the 57 participants with the deletion, patients were considered PPS(+) (*n* = 40) if they scored ≥3 in at least 1 visit on any of the subscales for PPSs. The remaining participants were considered PPS(−) (*n* = 17) [number of scans: PPS(+) = 101, PPS(−) = 47]. This criterion was chosen consistent with studies showing the clinical value of this threshold for determining the psychosis high-risk state (48). Given that frequency and intensity of PPSs are known to fluctuate considerably across time, to characterize atypical neurodevelopmental mechanisms that might contribute vulnerability to their emergence, a score ≥3 represents a more liberal threshold, which would essentially separate individuals who endorsed psychotic symptoms at some point from those who did not.

### Magnetic Resonance Imaging Acquisition and Processing

Resting-state functional magnetic resonance imaging (rs-fMRI) data were acquired using a 3T Siemens Trio (*n* = 220) and a 3T Siemens Prisma-fit (MAGNETOM Trio Upgrade) (*n* = 87) scanner (see Supplemental Material and Methods for details about the sequence parameters used). rs-fMRI data were processed using SPM12 (http://www.fil.ion.ucl.ac.uk/spm/) and DPARSF. Voxels were averaged using 200 cortical (49) and 16 bilateral subcortical (50) regions of interest (ROIs). A bandpass filter (0.01–0.1 Hz) was applied, resulting in resting-state functional time courses (RTFTCs) for each ROI. For each pair of RTFTCs, the Pearson correlation was computed, resulting in a 232 × 232 functional connectome. An explanation of the rs-fMRI processing pipeline is provided in Supplemental Material and Methods.

### Longitudinal Connectome Fingerprinting Framework

**Figure 1:**
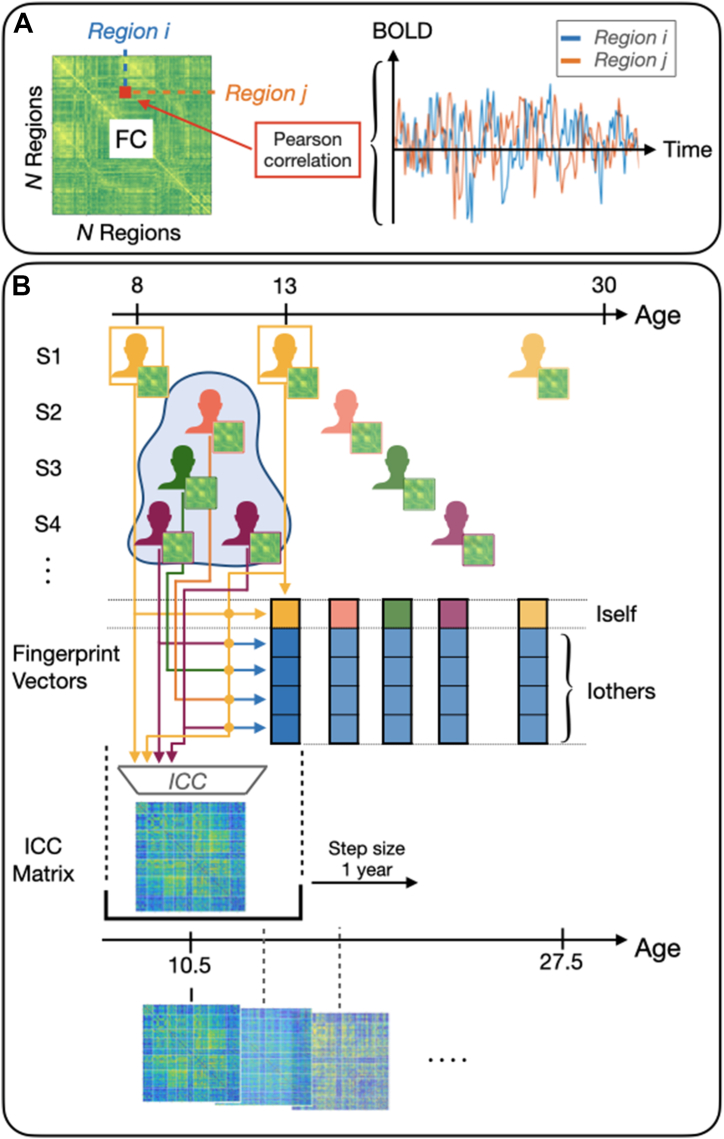
Combining sliding window approach with connectome fingerprinting and intraclass correlation coefficient (ICC) toward longitudinal analysis. **(A)** Pearson correlation between pairs of blood oxygen level–dependent (BOLD) time courses (between 232 atlas regions), resulting in a functional connectome for each participant and each visit. **(B)** Sliding window methodology incorporating longitudinal connectome fingerprinting and dynamic ICC analysis; temporal interval spanned by a rectangular window (5 years window size). Each computation is repeated iteratively, shifting the window by 1 year every time, to generate subsamples falling within this time span. Each subsample was then applied on an adapted connectome fingerprinting methodology to compute participant level measures of Iself, Iothers, and identification rate. Participants within each subsample with 2 visits were used to compute the edgewise ICC matrix. FC, functional connectivity.

To characterize developmental changes in functional connectome stability and individual distinctiveness, we implemented a longitudinal connectome fingerprinting framework combined with an age-based moving-window approach (Figure 1). This framework extends traditional test-retest fingerprinting (17) to longitudinal datasets with varying numbers of scans per participant by treating consecutive visit pairs as the fundamental unit of analysis.

#### Longitudinal Unit of Analysis: Consecutive Visit-Pairs

Participants contributed between 2 and 5 rs-fMRI scans. For participants with >2 scans, connectome fingerprinting metrics were computed for all temporally adjacent visit pairs (e.g., visit 1–2, 2–3, 3–4), yielding multiple longitudinal instances per participant.

For each visit pair, functional connectomes from consecutive visits were compared to quantify within-subject similarity and between-subject distinctiveness. Metrics were therefore defined at the visit-pair level rather than averaged across scans. Each visit pair was assigned the participant’s age at the later visit for subsequent inclusion in age-based moving windows.

#### Longitudinal Connectome Fingerprinting Metrics

Fingerprinting metrics were computed independently for each consecutive visit pair as follows:

1. Iself (within-subject similarity): Iself was defined as the Pearson correlation between the functional connectomes from 2 consecutive visits of the same participant. Each participant contributed 1 Iself value per consecutive visit pair.
2. Iothers (between-subjects similarity): For each visit-pair, Iothers was defined as the set of Pearson correlations between the participant’s functional connectome at the later visit and the functional connectomes of all other participants whose scan ages fell within the age range spanned by the visit pair. This age-restricted comparison ensured that between-subjects similarity was evaluated among developmentally comparable scans. The maximum value was retained for identification.
3. IdRate: Identification success was evaluated at the visit-pair level. For a given visit pair, identification was considered successful (value = 1) if the Iself exceeded the maximum Iothers value; otherwise, identification was considered unsuccessful (value = 0).

Within each age window, IdRate was defined as the proportion of successful identifications across visit pairs.

#### Moving-Window Aggregation Across Development

To examine developmental trajectories of connectome fingerprinting metrics, visit-pair instances were grouped into overlapping age-based windows (5-year width, 1-year step). Assignment to windows was based on the participant’s age at the later visit of each consecutive visit pair, ensuring temporal alignment of longitudinal changes with developmental stage. Given the typical interscan interval in the sample (approximately 3–4 years), each participant could contribute at most 1 consecutive visit pair to any given age window, which ensured approximately independent participant contributions within each window. Thus, no additional participant-level filtering within windows was required.

Within each window, mean Iself, mean Iothers, and IdRate (defined as the proportion of visit-pair instances showing successful identification) were computed. Windows with insufficient numbers of contributing participants were excluded from further analyses.

#### Longitudinal ICC

To assess functional stability at the level of individual connections, we computed longitudinal ICCs. Edgewise ICCs were calculated across consecutive visit pairs that fell within the same age window, providing a complementary measure of temporal stability at the network level.

Whereas connectome fingerprinting metrics capture whole-pattern distinctiveness, ICC quantifies the consistency of individual functional connections across time, allowing assessment of maturational changes in connection-specific stability.

### Statistical Analysis

To assess group differences in developmental trajectories, linear mixed-effects models were applied separately to IdRate and whole brain–averaged ICCs. In each model, the dependent variable was the window-averaged metric. Fixed effects included group, age, age^2^, and group-by-age interaction terms, where age (and age^2^) corresponded to the center age of each moving window.

A random intercept for age window was included to account for between-window variability. Because adjacent age windows overlapped in time and shared contributing visit-pair instances, a first-order autoregressive (AR[1]) correlation structure was applied to model temporal dependencies across windows. False discovery rate correction was applied to control for multiple comparisons.

### Joint PCA of FC and Temporal Stability

To identify shared and distinct dimensions of functional connectome maturation, we performed joint PCA combining FC and ICC matrices from all age windows. This approach captures coordinated patterns of network organization and temporal stability across development rather than testing formal hypotheses.

Individual FC matrices were averaged within each age window. FC and ICC matrices were vectorized (upper triangle only) and *z* scored across edges to ensure comparable scaling. The resulting feature sets were concatenated to form a combined data matrix (age windows × measures) and subjected to PCA via singular value decomposition. Component loadings reflected each edge’s contribution to spatial (FC) and temporal (ICC) variance, while component scores represented the projection of each age window onto the latent axes. The first 3 components were retained. FC and ICC loadings were mapped back onto the connectome to identify dominant functional systems, and component scores were visualized across developmental windows to characterize trajectories.

To assess robustness, we performed bootstrap resampling of participant pairs within age windows (*n* = 1000). For each iteration, FC and ICC matrices were recomputed, and the full PCA pipeline was repeated. Resulting component scores were aligned to the original solution using Procrustes transformation. The distribution of aligned scores was used to construct 95% confidence ellipsoids for each group at each window, and group separation was quantified using Mahalanobis distances between centroids relative to combined radii.

## Results

### Fingerprinting Reveals Altered Maturation in 22q11

We examined developmental changes in functional connectome identifiability by modeling age-related trajectories of IdRate in individuals with 22q11.2DS grouped by attenuated psychosis symptoms [PPS(−) and PPS(+)] and HC participants.

**Figure 2:**
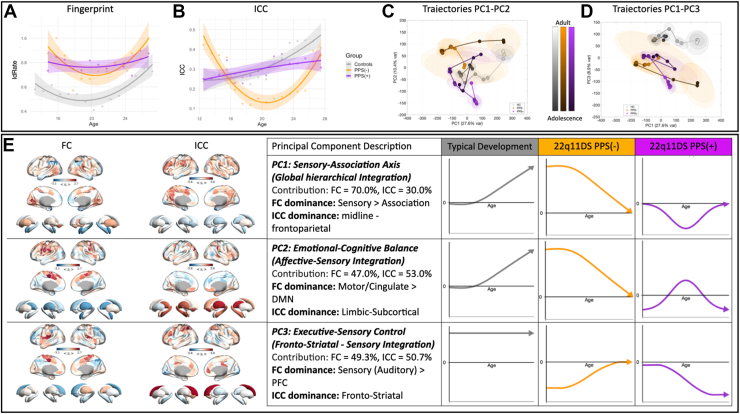
**(A)** Whole-brain averaged trajectories of identification rate (IdRate) in individuals without positive psychotic symptoms [PPS(−)], individuals with PPSs [PPS(+)], and control participants. **(B)** Whole-brain averaged trajectories of intraclass correlation coefficients (ICCs) in PPS(−) individuals, PPS(+) individuals, and control participants. **(C, D)** Developmental trajectories projected onto the principal component (PC) space: PC1-PC2 and PC1-PC3 for PPS(−) individuals, PPS(+) individuals, and control participants. Colors follow a gradient from adolescence (darker tones) to adulthood (brighter tones). Semitransparent ellipsoids represent 95% confidence regions derived from bootstrap resampling, illustrating the variability and separability of group trajectories across age windows. **(E)** Left: functional connectivity (FC) loadings and ICC loadings of the 3 principal components, reconstructed to its respective connectomes and subsequently nodal averaged into node measure displayed as brain plots. Middle: description of the dominant FC and ICC patterns associated with each PC. Right: schematic illustration summarizing the developmental trajectories along the 3 PCs for each group. 22q11DS, 22q11 deletion syndrome; DMN, default mode network; PFC, prefrontal cortex.

HC participants showed relatively low but stable IdRate across adolescence, with a mild decrease peaking near age 18 followed by a gradual adult increase (Figure 2A). In contrast, both deletion groups exhibited overall higher IdRate throughout development. PPS(−) individuals demonstrated an initial high start, a large decrease with a nadir around age 20, and a subsequent increase, while PPS(+) individuals expressed a similar but more flattened shape. As group effects included age interactions, regression coefficients in Table 2 reflect differences at the reference age (age = 0), while Table 3 shows age-adjusted pairwise comparisons across the observed range. Comparisons confirmed significant differences between HC participants and PPS(−) individuals and HC participants and PPS(+) individuals (Tukey-adjusted *p* < .0001), with no difference between the PPS groups (*p* = .27).

**Table 2:** Fixed-Effects Estimates From the Linear Mixed-Effects Model Examining Identification Rate Differences Across Groups and Age

Effect	Estimate	SE	*t*_37_	*p* Value
(Intercept)	1.3029	0.2039	6.39	<.0001
Group: PPS(−)	2.5260	0.7177	3.52	.0012
Group: PPS(+)	0.1544	0.7177	0.22	.831
Age	−0.0851	0.0218	−3.90	.0004
Age^2^	0.00230	0.00055	4.20	.0002
Group [PPS(−)] × Age	−0.2220	0.0711	−3.12	.0035
Group [PPS(+)] × Age	0.0148	0.0711	0.21	.837
Group [PPS(−)] × Age^2^	0.00521	0.00171	3.04	.0043
Group [PPS(+)] × Age^2^	−0.00053	0.00171	−0.31	.760

PPS, positive psychotic symptoms.

**Table 3:** Age-Adjusted Pairwise Comparisons of Estimated Marginal Means (Bonferroni Adjusted)

Contrast	Estimate	SE	*t*_37_ Ratio	*p* Value
Control − PPS(−)	−0.2864	0.0303	−9.464	<.0001
Control − PPS(+)	−0.2251	0.0303	−7.436	<.0001
PPS(−) − PPS(+)	0.0614	0.0351	1.746	.2673

PPS, positive psychotic symptoms.

**Table 4:** Fixed-Effects Estimates From the Linear Mixed-Effects Model Examining Intraclass Correlation Differences Across Groups and Age

Effect	Estimate	SE	*t*_39_	*p* Value
(Intercept)	0.4128	0.2256	1.83	.075
Group: PPS(–)	1.9278	0.3191	6.04	<.0001
Group: PPS(+)	−0.2988	0.3191	−0.94	.355
Age	−0.0266	0.0234	−1.14	.262
Age^2^	0.00105	0.00058	1.80	.079
Group [PPS(−)] × Age	−0.1883	0.0331	−5.69	<.0001
Group [PPS(+)] × Age	0.0390	0.0331	1.18	.245
Group [PPS(−)] × Age^2^	0.00417	0.00082	5.08	<.0001
Group [PPS(+)] × Age^2^	−0.00120	0.00082	−1.45	.154

PPS, positive psychotic symptoms.

**Table 5:** Age-Adjusted Pairwise Comparisons of Estimated Marginal Means (Bonferroni Adjusted)

Contrast	Estimate	SE	*t*_39_ Ratio	*p* Value
Control − PPS(−)	0.0800	0.0155	5.150	<.0001
Control − PPS(+)	0.0218	0.0155	1.402	.5066
PPS(−) − PPS(+)	−0.0582	0.0155	−3.748	.0017

PPS, positive psychotic symptoms.

### ICC Corroborates Reduced Maturational Stability in Deletion Carriers

The dynamic stability of FC (ICC) largely paralleled the IdRate trajectories (Figure 2B). HC participants showed an increasing profile occurring slightly earlier than the IdRate trajectory. PPS(−) individuals showed a similar shape as the IdRate curve, whereas PPS(+) individuals exhibited a flatter trajectory with reduced age-related variation. The HC versus PPS(−) contrast remained significant after correction (*p* < .0001); HC versus PPS(+) did not (*p* = .51). There was a significant difference between the PPS groups (*p* = .0017). As for IdRate, the coefficients in Table 4 describe differences at the reference age, while Table 5 reports age-adjusted pairwise comparisons of estimated marginal means across the observed age range.

### PCA Identifies 3 Orthogonal Axes of Connectome Maturation

To identify connectivity patterns underlying global effects, we conducted PCA on FC and ICC matrices across all participants and time points (variance explained = 46.9%). Loadings were decomposed into FC and ICC contributions, focusing on the first 3 orthogonal dimensions of connectome maturation (Figure 2E). These components captured complementary aspects of developmental organization, guided by the spatial distribution of FC and ICC loadings mapped onto the connectome. Three dominant axes emerged: PC1, sensory-association (global hierarchical integration); PC2, emotional-cognitive balance (affective-sensory integration); and PC3, executive-sensory control (frontostriatal-sensory integration). Trajectories along these axes showed distinct patterns between HC participants, PPS(−) individuals, and PPS(+) individuals, reflecting graded alterations in temporal coordination and network integration.

Bootstrap analysis confirmed robust group separation across most developmental windows (Figure 2C, D), indicating stable and reproducible differences in connectome maturation. Partial overlap was observed only at age 16.5 [HC vs. PPS(+)] and in the final 2 windows [26.5 and 27.5 years; PPS(−) vs. PPS(+)] (see the Supplement).

#### PC1: Sensory-Association Axis

HC participants exhibited an overall linear increase in PC1 scores from adolescence into adulthood, presenting progressive strengthening and coordination of sensory-association network integration. PPS(−) individuals exhibited a decline over time, picturing strong reorganization but preserved directional maturation. In contrast, PPS(+) trajectories were nonmonotonic, showing an initial increase followed by a decrease and a return toward start point in adulthood.

#### PC2: Emotional-Cognitive Balance

HC participants transitioned from slightly negative values during adolescence to positive values in adulthood, presenting a normative shift from sensorimotor dominance to mature affective-cognitive balance. PPS(−) individuals began above 0 but trended downward toward HC participants in adulthood, indicating delayed alignment with typical affective-cognitive regulation. PPS(+) individuals again showed an irregular trajectory, repeatedly shifting across 0 and failing to stabilize a dominant organizational mode.

#### PC3: Executive-Sensory Control

HC participants maintained consistently positive PC3 scores, presenting stable and flexible executive integration with sensory systems. PPS(−) trajectories began negative and moved toward 0 over time, suggesting compensatory strengthening of executive integration. In contrast, PPS(+) trajectories declined into negative values, indicating progressive decoupling of prefrontal control from sensory systems and increasing rigidity of functional organization with age.

### Group-Specific Trajectories in Multidimensional Maturation Space

Integrating all 3 components in a common 3-dimensional latent space revealed distinct and statistically separable developmental trajectories across groups. HC participants followed a smooth and directed maturational trajectory through PCA space, reflecting coordinated large-scale integration. PPS(−) individuals exhibited a globally shifted but structured trajectory, maintaining partial alignment with normative development, particularly along PC3. In contrast, PPS(+) individuals showed a disorganized and nonmonotonic trajectory, lacking a coherent developmental direction in the PC1-PC2 plane and exhibiting progressive divergence along PC3.

Bootstrap-derived confidence ellipsoids confirmed that these trajectories remain significantly separated across development, supporting the presence of stable group differences in the organization of FC and temporal stability.

## Discussion

In this study, we examined the longitudinal development of functional connectome uniqueness in individuals with 22q11DS stratified by psychotic symptom presence across a developmental span from early adolescence into adulthood. By integrating connectome fingerprinting, edgewise stability (ICC), and a new joint FC-ICC PCA, we identified distinct developmental signatures underlying potential network-level mechanisms of resilience versus vulnerability to psychosis.

### From a Period of Rapid Changes in Adolescence to Stabilization During Adulthood

Typically developing individuals exhibited a fingerprint trajectory characterized by reduced identifiability during adolescence followed by a steady increase in adulthood. This pattern is consistent with developmental models describing adolescence as a period of heightened network variability, enabling greater functional flexibility that facilitates large-scale pruning and reorganization and eventually consolidating into a stable adult connectome (24,51,52). This neurotypical developmental trajectory was expressed across PCs of the joint FC-ICC space. PC1 expressed an overall increase in hierarchical connectivity strength and temporal stability, indicating progressive stabilization of large-scale functional organization. PC2 captured a shift from early affective-sensorimotor predominance toward balanced affective-cognitive integration, consistent with the transition from emotion- and body-centered reactivity to higher-order regulatory control across adolescence (53). PC3 remained positive throughout development, indicating efficient executive-sensory integration and consistent top-down coordination (54, 55, 56). Together, these trajectories characterize an orderly developmental sequence: during adolescence, a transient reorganization of sensory and affective systems, followed by the consolidation of a stable, hierarchically integrated network architecture that supports mature cognitive-emotional and executive-sensory control (57). Importantly, these trajectories were stable across bootstrap resampling, indicating that the observed developmental patterns reflect robust features of connectome maturation rather than sampling variability.

### Divergence in 22q11DS: Compensatory Versus Maladaptive Development

Although the most pronounced group-by-age interaction in the global fingerprinting and ICC measures was observed in the PPS(−) subgroup, the PCA trajectories revealed a different qualitative pattern distinguishing the PPS(+) from both HC and PPS(−) groups. Specifically, the PPS(−) group showed a marked but temporally structured developmental modulation of connectome stability, whereas the PPS(+) group was characterized by irregular and nondirectional developmental trajectories across the joint FC-ICC dimensions. PPS(−) individuals exhibited a pronounced drop in fingerprint accuracy during adolescence, followed by an increase in adulthood. This pattern was paralleled by an initial decrease followed by a moderate restoration of hierarchical stability (PC1) and a progressive increase toward executive control (PC3). Such a trajectory may reflect compensatory reorganization, whereby the system transiently destabilizes to regain adaptive integration between executive and sensory systems (58), potentially underpinning resilience to the development of psychotic symptoms despite overall reduced stability (59).

In contrast, PPS(+) participants followed a markedly different path. Their IdRate and ICC stability showed little evidence of age-related improvement. This shallow developmental profile is reflected in irregular trajectories in the PC1-PC2 space, indicating failures to consolidate straightforward global hierarchical integration (PC1) and affective-cognitive regulation (PC2). Although PCA provides a dimensional representation of these processes, bootstrap analyses confirmed that the observed trajectories are statistically stable and consistently separated across groups. Moreover, PC3 values, after an initially unstable course, became increasingly negative with age, diverging from the efficient executive-sensory integration observed in HC participants and PPS(−) individuals. The brain pattern of this negative shift reflects nonselective strengthening of executive-to-whole brain connectivity coupled with unstable sensory-attention connectivity. Rather than facilitating targeted top-down control as is observed in typical development, such a configuration implies excessive but poorly focused executive influence, aggravated by weakened large-scale integration and impaired emotional-cognitive balance.

This multiaxis developmental pattern observed in PPS(+) individuals is consistent with neurodevelopmental models of psychosis that emphasize dyscoordination between sensory, limbic, and executive systems. Aberrant interactions between these systems may degrade sensory specificity and self-monitoring processes, potentially fostering misattribution of internally generated experiences and increasing vulnerability to hallucinations and thought disturbance (60,61). Importantly, these interpretations regarding PPS(+) individuals remain speculative and should not be viewed as direct evidence of specific neurobiological mechanisms. Given that the strongest group-by-age effects in global fingerprinting and ICC measures were observed in PPS(−) individuals, the PPS(+) findings are better interpreted as qualitative trajectory differences within the joint FC-ICC space that are broadly consistent with models of psychosis vulnerability rather than definitive mechanistic signatures.

### Psychosis Risk: A Loss of Developmental Timing and Directionality

On the one hand, the patterns obtained by the joint FC-ICC components capture the neural correlates of complex behavioral development with regard to psychotic symptoms. On the other hand, they may reflect underlying neurobiological processes that sculpt large-scale brain organization. For example, during typical maturation, progressive myelination and synaptic pruning drive both the strengthening of long-range integration and the reduction of redundant connections, producing increasingly selective and efficient connectivity profiles (43,62). These processes jointly enhance the stability of functional communication (indexed by ICC) and strengthen relevant coactivation (reflected in FC loadings).

The irregular trajectories observed in PPS(+) individuals across both PC1-PC2 and PC1-PC3 spaces may indicate alterations in developmental organization rather than a simple delay or attenuation of maturation. This interpretation is supported by the bootstrap analysis, which demonstrated consistent multivariate separation of PPS(+) individuals from both HC participants and PPS(−) individuals across nearly all developmental windows. In typical and resilient trajectories, these axes seem to evolve in clearer, directed paths, indicating coordinated large-scale processes, such as pruning, myelination, and circuit specialization, which follow temporally ordered phases. In contrast, the irregular pattern in PPS(+) individuals suggests reduced coherence in the unfolding of these developmental processes, consistent with a trajectory that does not settle into a stable maturational direction within the observed age range. Such instability may be compatible with reduced coordination of neurodevelopmental processes, such as loss of temporal coordination in the interaction of cellular and circuit-level processes (27).

Prior studies have suggested that chronic or intermittent neuroinflammatory states can alter glial and astrocytic activity, thereby distorting the timing and selectivity of key maturational processes (63).

In 22q11DS, vulnerabilities in blood-brain barrier integrity (64) and cellular metabolism (65, 66, 67, 68) may heighten susceptibility to neuroinflammation, which can disrupt glial regulation and thus the timing of maturational processes (69,70). These perturbations have been linked to altered glutamatergic signaling, reduced NMDA receptor function (71,72), and impaired development of parvalbumin interneurons (73,74), resulting in a shift in excitation-inhibition balance. This imbalance may destabilize large-scale network coordination, offering a mechanistic route to the functional dysconnectivity associated with psychosis (9,75).

Given this literature, the irregular and desynchronized maturation pattern observed in the PPS(+) group may represent a macroscopic signature of disrupted developmental coordination. Rather than reflecting a simple delay in maturation, the nondirected PCA trajectories suggest a loss of temporal alignment across large-scale network processes. In this framework, typical and resilient trajectories appear to follow relatively directed developmental paths, whereas PPS(+) may reflect a breakdown in the coordination of processes shaping network maturation.

Thus, the nondirected PC trajectories in PPS(+) individuals may reflect altered large-scale developmental organization, although the precise biological mechanisms underlying these patterns remain unclear.

These findings underscore how disrupted developmental timing, rather than static deficits, may constitute a core neurobiological signature of psychosis risk. Together, these observations suggest 2 distinct modes of developmental deviation in 22q11DS: a structured but delayed reorganization in PPS(−) individuals, potentially reflecting compensatory plasticity, and a loss of directional maturation in PPS(+) individuals, characterized by irregular trajectories across multiple network dimensions.

### Limitations

Several limitations of the current work should be acknowledged. Although cognitive and psychiatric factors were assessed (Table 1), the study was not powered to disentangle effects of specific comorbidities from primary diagnostic group differences. Medication status was also not modeled due to heterogeneity in type, dosage, and timing, so medication-related effects on FC and stability cannot be excluded. Future studies should address this using specifically placebo-controlled study designs. Finally, although 22q11.2DS is associated with altered brain volume, we did not include volumetric covariates, as analyses focused on within-subject similarity of normalized FC. While this reduces sensitivity to structural differences, indirect morphological effects cannot be ruled out. Multimodal studies are needed to examine this more directly.

### Conclusions

By integrating longitudinal FC and ICC data within a shared PCA framework, this study moves connectome fingerprinting beyond identifiability toward a multidimensional model of functional brain maturation. The decomposition revealed separable axes that encode both spatial and temporal aspects of development: PC1 captured global hierarchical integration, PC2 reflected shifts in emotional-cognitive balance, and PC3 indexed the integration of executive and sensory systems. Mapping longitudinal trajectories along these axes revealed distinct developmental patterns that differentiate resilience from vulnerability to psychosis. These patterns were further supported by bootstrap-derived confidence ellipsoids, demonstrating robust multivariate separation between groups across development. Importantly, these findings demonstrate that connectome individuality alone is not sufficient to explain developmental outcome. Instead, the direction and temporal organization of network maturation appear to be critical for determining whether developmental processes unfold adaptively or become destabilized. Together, these findings help extend functional fingerprinting toward a more dimensional neurodevelopmental framework, suggesting a possible route for relating trajectories of brain network maturation to emerging clinical risk and resilience.

Future research should broaden this developmental fingerprinting approach to nonsyndromic high-risk groups and incorporate models that distinguish between normative instability (reflecting adaptive pruning) and pathological disorganization. Furthermore, clinical longitudinal cohorts with shorter age intervals between visits could increase the sensitivity of our longitudinal fingerprinting by using a window size smaller than 5 years. Moreover, interventions targeting the timing and flexibility of key developmental periods, whether behavioral, pharmacological, or neuromodulatory, could help redirect atypical developmental trajectories toward more adaptive configurations. Finally, the pronounced divergence in fingerprint and ICC patterns already observable at age 12.5 between PPS(−) and PPS(+) individuals underscores the urgency of shifting neuroimaging studies further upstream into early and middle childhood. Capturing these windows before the brain either reorganizes adaptively or prematurely locks into maladaptive configurations may prove critical for early detection and prevention in neuropsychiatric risk populations.

## Supplementary Material

Supplemental Material and Methods, Results, Figures S1–S14, and Tables S1–S6

Key Resources Table
