# Supplementary material for: Developmental Brain Network Trajectories Differentiate Resilience and Vulnerability to Psychosis in 22q11.2 Deletion Syndrome
Source: Biol Psychiatry Glob Open Sci. 2026 Jun 10;6(5):100768. doi: 10.1016/j.bpsgos.2026.100768 (PMC13400943; doi:10.1016/j.bpsgos.2026.100768)
Supplement: Supplemental Material and Methods, Results, Figures S1–S14, and Tables S1–S6 [file mmc1.pdf]

SUPPLEMENTARY INFORMATION

Developmental Brain Network Trajectories Differentiate Resilience and Vulnerability to Psychosis in 22q11.2 Deletion Syndrome

Forrer *et al.*

Supplemental Material and Methods

Participants

This study was carried out within one of the largest prospective on-going longitudinal study on 22q11DS worldwide, conducted in Geneva, Switzerland also described in previous literature [1], [2], [3]. Recruitment was performed through patient associations and word of mouth. Subjects are followed up approximately every 3 years. At each assessment subjects undergo the acquisition of neuroimaging data, as well as clinical and cognitive testing. In the current study, the inclusion criteria for the longitudinal analyses comprised the presence of fMRI data, in good condition, acquired exclusively with a 3T scanner. Diagnosis of 22q11.2 deletion was confirmed following a quantitative fluorescent polymerase chain reaction performed in the Department of Medical Genetics in Geneva. HCs were enrolled from unaffected siblings of the patients or through an open call to the Geneva State School system in Switzerland.

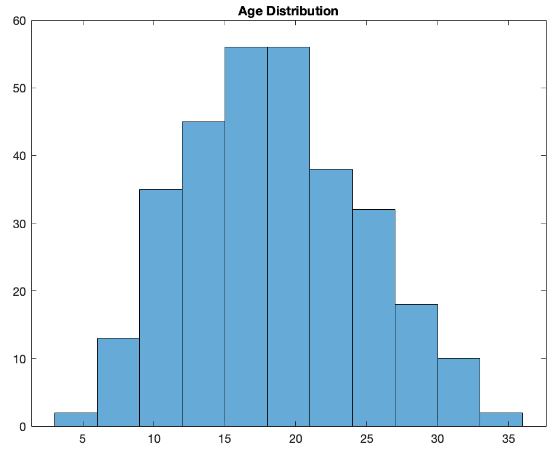

Figure S1: Histogram of age distribution of all subjects and visits

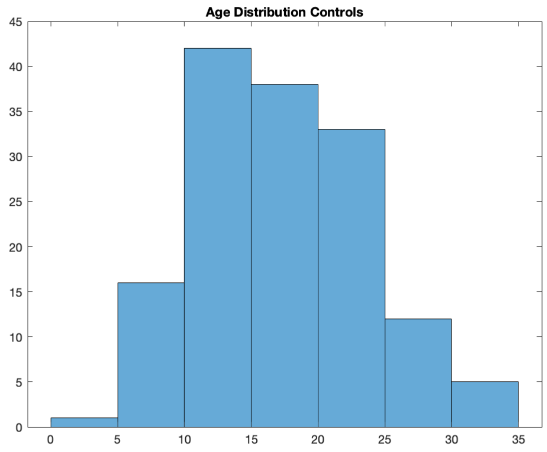

Figure S2 Histogram of age distribution of controls

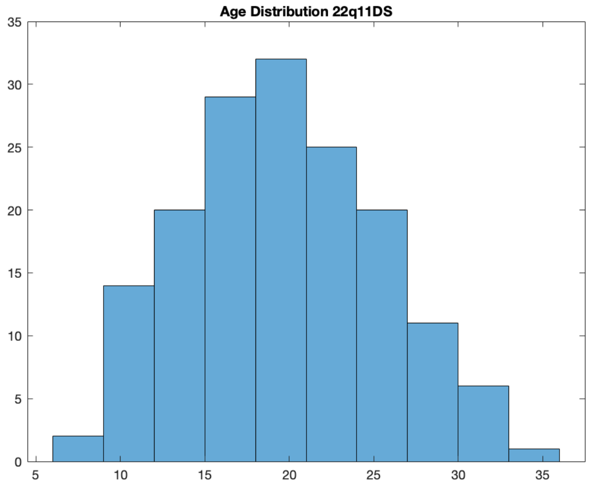

FigureS3: Histogram of age distribution of 22q11DS subjects

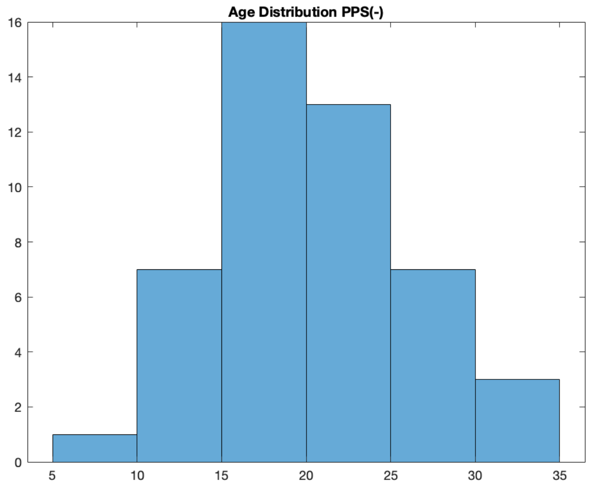

Figure S4: Histogram of age distribution of PPS(-)

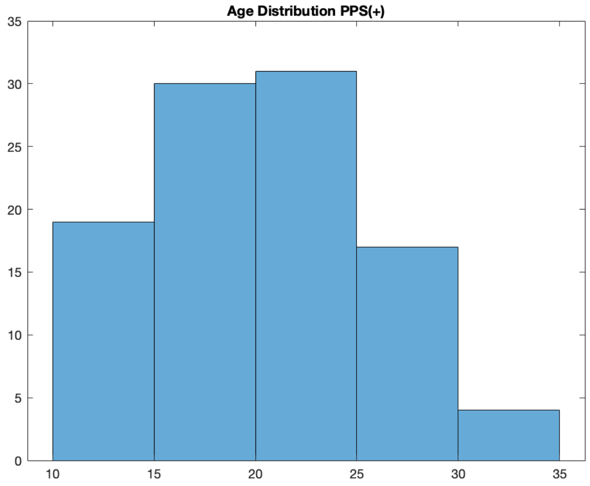

Figure S5: Histogram of age distribution of PPS(+)

MRI acquisition

fMRI images were acquired using a T2-weighted sequence (200 frames) with following parameters: acquisition matrix = 94×128, field of view = 96x128, voxel size = 1.84×1.84×3.2 mm^3, 38 axial slices, slice thickness = 3.2 mm, TR=2400 ms, TE=30 ms, flip angle=85°, phase encoding A >> P, descending sequential ordering, GRAPPA acceleration mode with factor PE=2.

fMRI processing

rs-fMRI data were processed using SPM12 (Wellcome Trust Centre for Neuroimaging, London, UK: http://www.fil.ion.ucl.ac.uk/spm/) and the Data Processing Assistant for rs-fMRI (DPARSF). For each participant, functional images were realigned over time and spatially smoothed with an isotropic Gaussian kernel of 6 mm full width at half maximum (FWHM). Subsequently, anatomical images were coregistered to the functional space and segmented with the SPM12 Segmentation algorithm [4]. Voxels were averaged using 200 cortical [5] and 16 bilateral subcortical [6] regions of interest (ROIs). Using the study-specific DARTEL template [7], ROIs were spatially transformed into the individual subject space and resliced to fMRI resolution to parcellate the functional images into 232 ROIs. Nuisance variables were regressed out (6 head motion parameters + other 6, average cerebrospinal fluid, and white matter signal). The first five frames were excluded, and a bandpass filter (0.01 Hz - 0.1 Hz) was applied. resulting in resting-state functional time courses (RSFTC) for each ROI. Motion scrubbing [8] was finally applied for the correction of motion artifacts based on the framewise displacement (FD), which is defined as the sum of the absolute values of the six realignment parameters. Volumes with FD larger than 0.5 mm were excluded together with the previous and two consecutive time points. At last, regional RSFTC were z-scored.

Supplemental Results

Number of Subjects within each window for Iself, Iothers and idRate

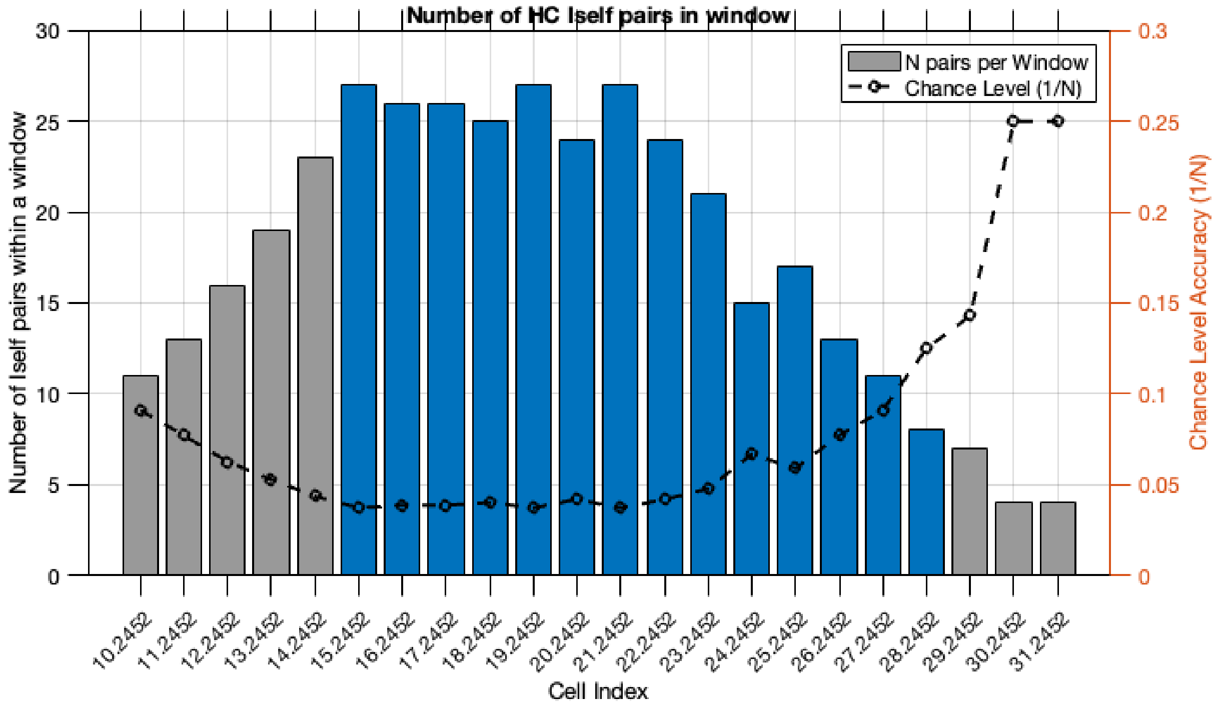

Figure S6: Histogram of number Healthy Controls within each window, Black: Chance level prediction accuracy, Blue bars: windows used for the combined FC-ICC PCA

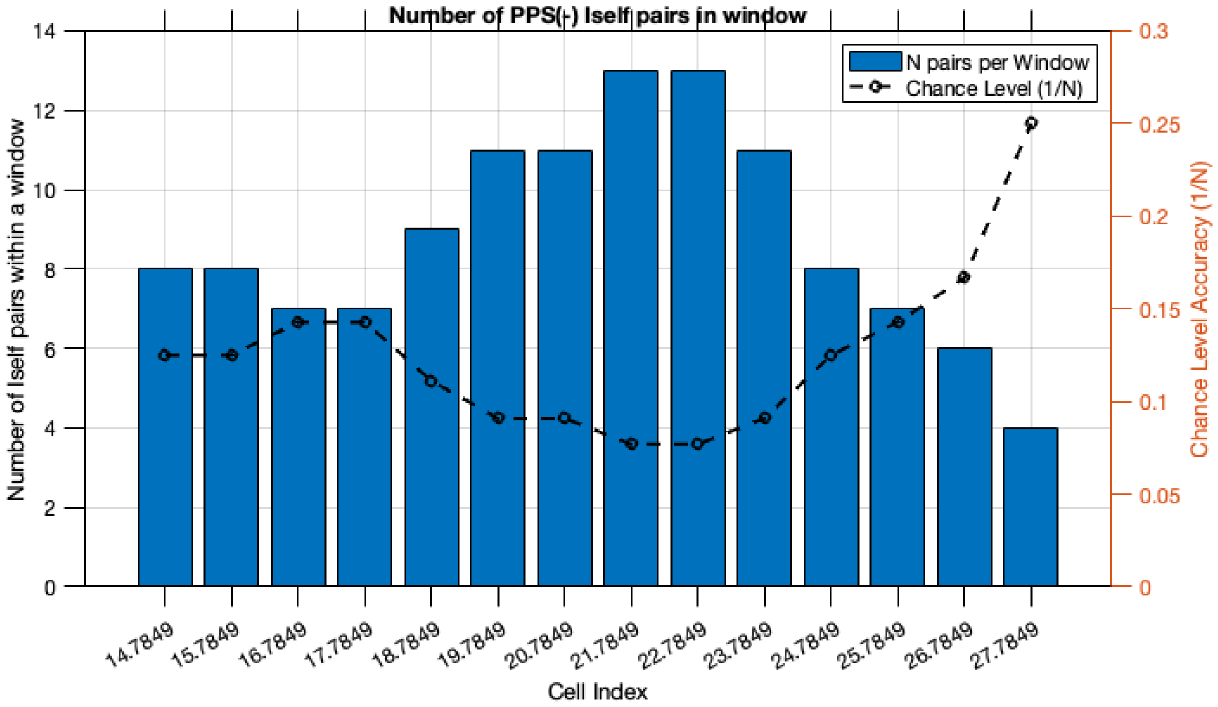

Figure S7: Histogram of number PPS(-) within each window, Black: Chance level prediction accuracy

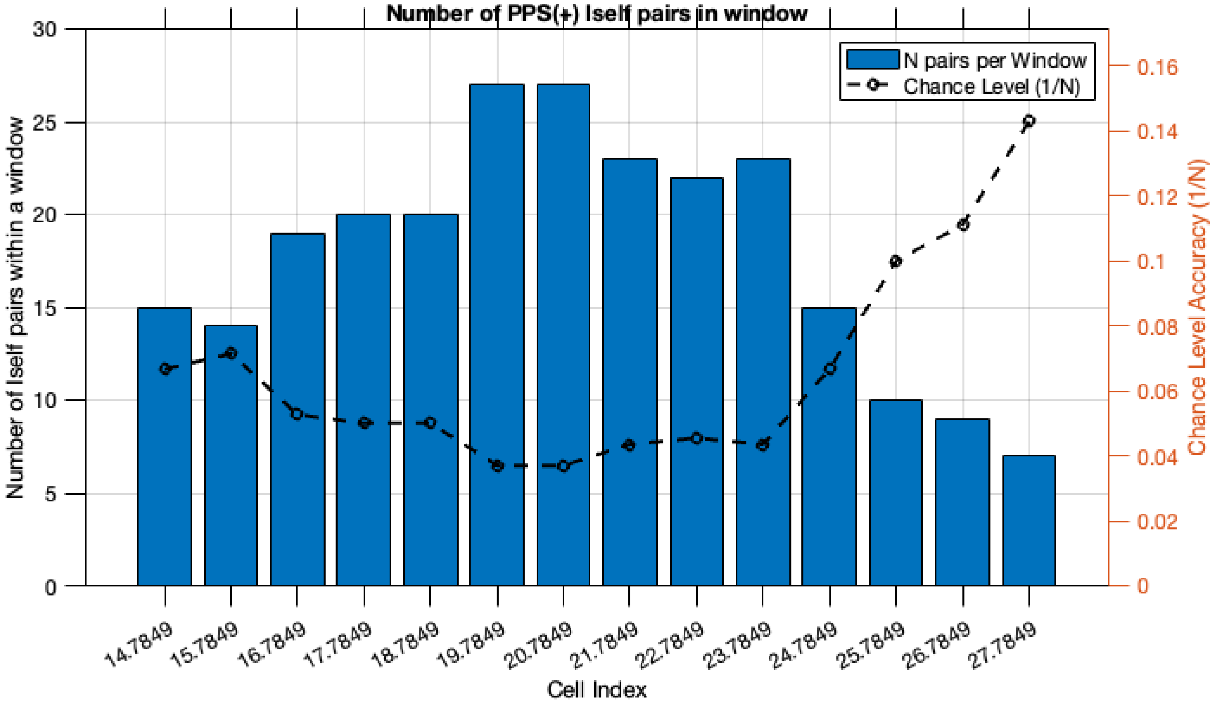

Figure S8: Histogram of number PPS(+) within each window, Black: Chance level prediction accuracy

Number of Subject Visit-Pairs within a window for ICC analysis

Window Nbr	Age Window Center	N pairs Controls	N pairs PPS(-)	N pairs PPS(+)
1	12.5	23	5	9
2	13.5	27	6	12
3	14.5	28	8	14
4	15.5	27	6	13
5	16.5	25	8	19
6	17.5	27	7	20
7	18.5	26	10	20
8	19.5	25	11	25
9	20.5	22	12	26
10	21.5	20	13	23
11	22.5	15	13	21
12	23.5	15	10	22
13	24.5	13	8	16
14	25.5	10	8	12
15	26.5	7	6	10
16	27.5	6	5	9

Table S1: Number of Subjects Visit-Pairs per window used for the intraclass correlation analysis.

Model output of mixed effect models for idRate

Age	Contrast	Estimate	SE	df	t-ratio	p-value
12	Controls - (PPS-)	-2.0733	0.5850	37	-3.545	0.0033
12	Controls - (PPS+)	-0.1084	0.5850	37	-0.185	1.0000
12	(PPS-) - (PPS+)	1.9649	0.7910	37	2.483	0.0530
13	Controls - (PPS-)	-1.8513	0.5140	37	-3.603	0.0028
13	Controls - (PPS+)	-0.1231	0.5140	37	-0.240	1.0000
13	(PPS-) - (PPS+)	1.7282	0.6960	37	2.485	0.0529
14	Controls - (PPS-)	-1.6293	0.4430	37	-3.680	0.0022
14	Controls - (PPS+)	-0.1379	0.4430	37	-0.311	1.0000
14	(PPS-) - (PPS+)	1.4914	0.6000	37	2.486	0.0527
15	Controls - (PPS-)	-1.4073	0.3720	37	-3.785	0.0016
15	Controls - (PPS+)	-0.1527	0.3720	37	-0.411	1.0000
15	(PPS-) - (PPS+)	1.2547	0.5040	37	2.488	0.0524
16	Controls - (PPS-)	-1.1854	0.3010	37	-3.939	0.0010
16	Controls - (PPS+)	-0.1674	0.3010	37	-0.556	1.0000
16	(PPS-) - (PPS+)	1.0179	0.4090	37	2.490	0.0521
17	Controls - (PPS-)	-0.9634	0.2300	37	-4.185	0.0005
17	Controls - (PPS+)	-0.1822	0.2300	37	-0.791	1.0000
17	(PPS-) - (PPS+)	0.7812	0.3130	37	2.493	0.0518
18	Controls - (PPS-)	-0.7414	0.1600	37	-4.638	0.0001
18	Controls - (PPS+)	-0.1970	0.1600	37	-1.232	0.6769
18	(PPS-) - (PPS+)	0.5444	0.2180	37	2.495	0.0515
19	Controls - (PPS-)	-0.5194	0.0907	37	-5.729	<.0001
19	Controls - (PPS+)	-0.2117	0.0907	37	-2.335	0.0752
19	(PPS-) - (PPS+)	0.3077	0.1240	37	2.483	0.0530
20	Controls - (PPS-)	-0.2974	0.0318	37	-9.357	<.0001
20	Controls - (PPS+)	-0.2265	0.0318	37	-7.125	<.0001
20	(PPS-) - (PPS+)	0.0709	0.0380	37	1.868	0.2090
21	Controls - (PPS-)	-0.0754	0.0626	37	-1.205	0.7077
21	Controls - (PPS+)	-0.2413	0.0626	37	-3.853	0.0013
21	(PPS-) - (PPS+)	-0.1658	0.0766	37	-2.165	0.1107

22	Controls - (PPS-)	0.1465	0.1300	37	1.125	0.8029
22	Controls - (PPS+)	-0.2560	0.1300	37	-1.966	0.1703
22	(PPS-) - (PPS+)	-0.4026	0.1690	37	-2.379	0.0678
23	Controls - (PPS-)	0.3685	0.2000	37	1.840	0.2213
23	Controls - (PPS+)	-0.2708	0.2000	37	-1.352	0.5535
23	(PPS-) - (PPS+)	-0.6393	0.2640	37	-2.421	0.0615
24	Controls - (PPS-)	0.5905	0.2710	37	2.180	0.1071
24	Controls - (PPS+)	-0.2856	0.2710	37	-1.054	0.8959
24	(PPS-) - (PPS+)	-0.8761	0.3590	37	-2.438	0.0591
25	Controls - (PPS-)	0.8125	0.3420	37	2.378	0.0681
25	Controls - (PPS+)	-0.3003	0.3420	37	-0.879	1.0000
25	(PPS-) - (PPS+)	-1.1128	0.4550	37	-2.446	0.0579
26	Controls - (PPS-)	1.0345	0.4130	37	2.507	0.0501
26	Controls - (PPS+)	-0.3151	0.4130	37	-0.764	1.0000
26	(PPS-) - (PPS+)	-1.3495	0.5500	37	-2.452	0.0572
27	Controls - (PPS-)	1.2564	0.4840	37	2.598	0.0402
27	Controls - (PPS+)	-0.3298	0.4840	37	-0.682	1.0000
27	(PPS-) - (PPS+)	-1.5863	0.6460	37	-2.455	0.0567
28	Controls - (PPS-)	1.4784	0.5550	37	2.665	0.0340
28	Controls - (PPS+)	-0.3446	0.5550	37	-0.621	1.0000
28	(PPS-) - (PPS+)	-1.8230	0.7420	37	-2.458	0.0564

Table S2: Age-specific pairwise group contrasts for idRate Estimated marginal means were derived at each integer age from 12 to 28 years. Bonferroni correction applied for 3 pairwise comparisons per age.

Model output of mixed effect models for ICC

Age	Contrast	Estimate	SE	df	t-ratio	p-value
12	Controls - (PPS-)	-1.4380	0.2670	39	-5.381	<.0001
12	Controls - (PPS+)	0.3375	0.2670	39	1.263	0.6426
12	(PPS-) - (PPS+)	1.7755	0.2670	39	6.643	<.0001
13	Controls - (PPS-)	-1.2497	0.2340	39	-5.335	<.0001
13	Controls - (PPS+)	0.2984	0.2340	39	1.274	0.6307
13	(PPS-) - (PPS+)	1.5481	0.2340	39	6.609	<.0001
14	Controls - (PPS-)	-1.0614	0.2010	39	-5.273	<.0001
14	Controls - (PPS+)	0.2594	0.2010	39	1.289	0.6153
14	(PPS-) - (PPS+)	1.3208	0.2010	39	6.562	<.0001
15	Controls - (PPS-)	-0.8731	0.1680	39	-5.187	<.0001
15	Controls - (PPS+)	0.2203	0.1680	39	1.309	0.5946
15	(PPS-) - (PPS+)	1.0934	0.1680	39	6.496	<.0001
16	Controls - (PPS-)	-0.6847	0.1350	39	-5.056	<.0001
16	Controls - (PPS+)	0.1813	0.1350	39	1.339	0.5654
16	(PPS-) - (PPS+)	0.8660	0.1350	39	6.395	<.0001
17	Controls - (PPS-)	-0.4964	0.1030	39	-4.836	0.0001
17	Controls - (PPS+)	0.1422	0.1030	39	1.386	0.5212
17	(PPS-) - (PPS+)	0.6386	0.1030	39	6.222	<.0001
18	Controls - (PPS-)	-0.3081	0.0701	39	-4.393	0.0002
18	Controls - (PPS+)	0.1032	0.0701	39	1.471	0.4479
18	(PPS-) - (PPS+)	0.4113	0.0701	39	5.864	<.0001
19	Controls - (PPS-)	-0.1198	0.0386	39	-3.104	0.0106
19	Controls - (PPS+)	0.0641	0.0386	39	1.662	0.3137
19	(PPS-) - (PPS+)	0.1839	0.0386	39	4.766	0.0001
20	Controls - (PPS-)	0.0685	0.0157	39	4.365	0.0003
20	Controls - (PPS+)	0.0251	0.0157	39	1.597	0.3552
20	(PPS-) - (PPS+)	-0.0435	0.0157	39	-2.768	0.0257
21	Controls - (PPS-)	0.2569	0.0345	39	7.442	<.0001
21	Controls - (PPS+)	-0.0140	0.0345	39	-0.405	1.0000
21	(PPS-) - (PPS+)	-0.2708	0.0345	39	-7.847	<.0001

22	Controls - (PPS-)	0.4452	0.0658	39	6.771	<.0001
22	Controls - (PPS+)	-0.0530	0.0658	39	-0.806	1.0000
22	(PPS-) - (PPS+)	-0.4982	0.0658	39	-7.577	<.0001
23	Controls - (PPS-)	0.6335	0.0982	39	6.451	<.0001
23	Controls - (PPS+)	-0.0921	0.0982	39	-0.938	1.0000
23	(PPS-) - (PPS+)	-0.7256	0.0982	39	-7.389	<.0001
24	Controls - (PPS-)	0.8218	0.1310	39	6.276	<.0001
24	Controls - (PPS+)	-0.1311	0.1310	39	-1.001	0.9686
24	(PPS-) - (PPS+)	-0.9530	0.1310	39	-7.277	<.0001
25	Controls - (PPS-)	1.0102	0.1640	39	6.166	<.0001
25	Controls - (PPS+)	-0.1702	0.1640	39	-1.039	0.9161
25	(PPS-) - (PPS+)	-1.1803	0.1640	39	-7.204	<.0001
26	Controls - (PPS-)	1.1985	0.1970	39	6.090	<.0001
26	Controls - (PPS+)	-0.2092	0.1970	39	-1.063	0.8827
26	(PPS-) - (PPS+)	-1.4077	0.1970	39	-7.154	<.0001
27	Controls - (PPS-)	1.3868	0.2300	39	6.036	<.0001
27	Controls - (PPS+)	-0.2483	0.2300	39	-1.081	0.8596
27	(PPS-) - (PPS+)	-1.6351	0.2300	39	-7.116	<.0001
28	Controls - (PPS-)	1.5751	0.2630	39	5.994	<.0001
28	Controls - (PPS+)	-0.2873	0.2630	39	-1.093	0.8427
28	(PPS-) - (PPS+)	-1.8625	0.2630	39	-7.088	<.0001

Table S3: Age-specific pairwise group contrasts for ICC. Estimated marginal means were derived at each integer age from 12 to 28 years. Bonferroni correction applied for 3 pairwise comparisons per age.

Overview of all FC matrices for each window

	Controls	PPS(-)	PPS(+)
Age 13-14	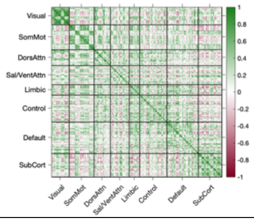		
Age 14-15	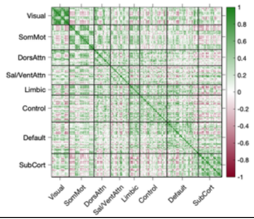	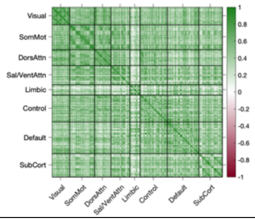	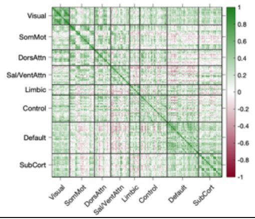
Age 15-16	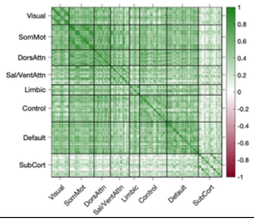	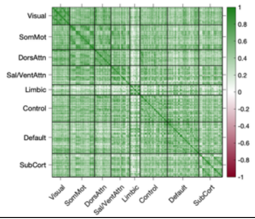	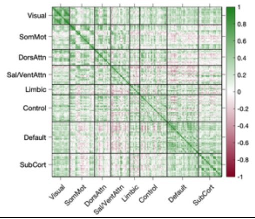
Age 16-17	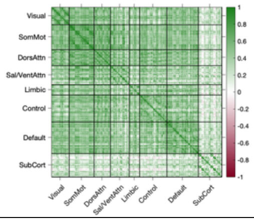	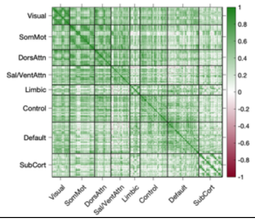	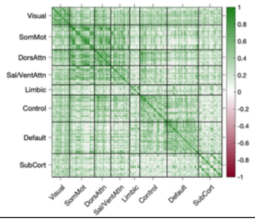
Age 17-18	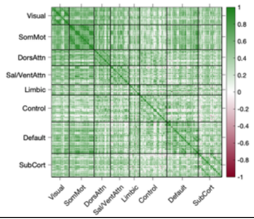	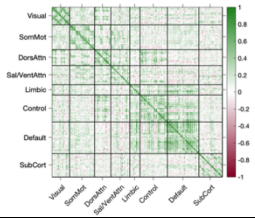	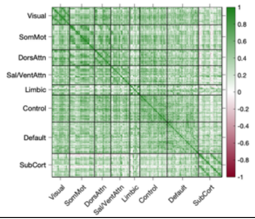
Age 18-19	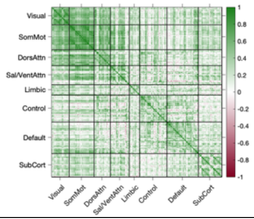	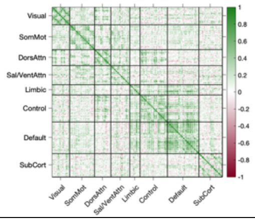	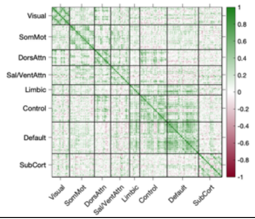
Age 19-20	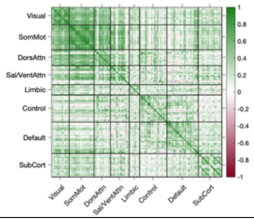	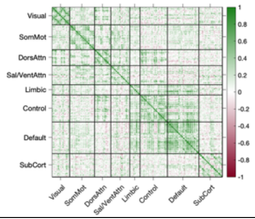	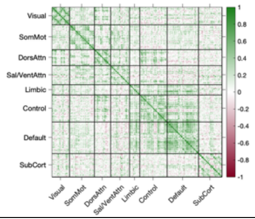

Age 20-21	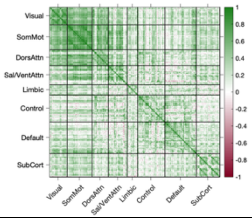	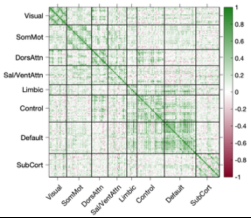	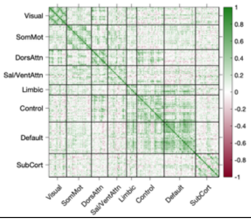
Age 21-22	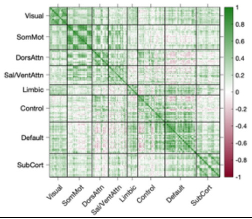	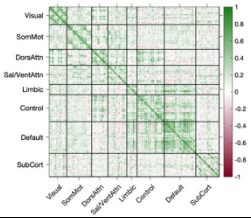	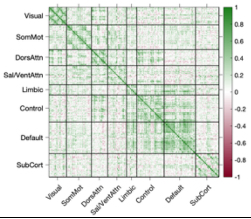
Age 22-23	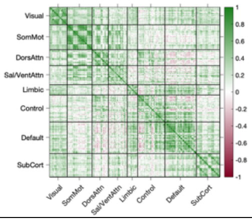	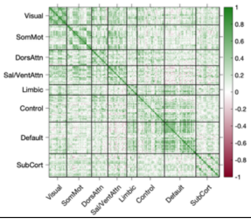	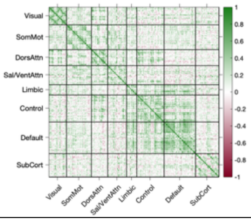
Age 23-24	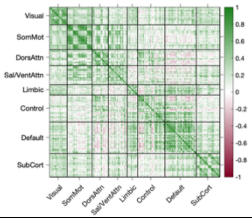	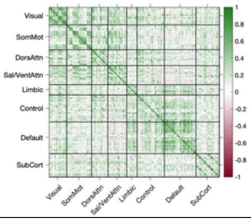	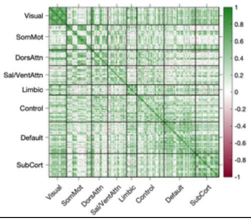
Age 24-25	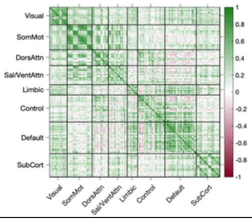	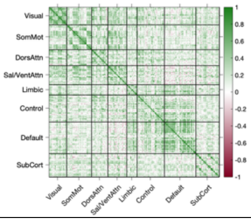	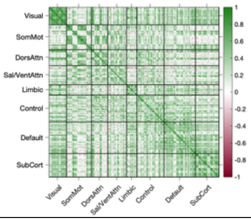
Age 25-26	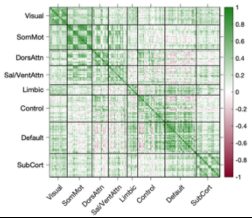	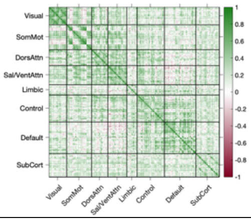	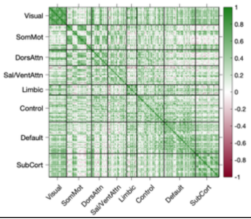
Age 26-27	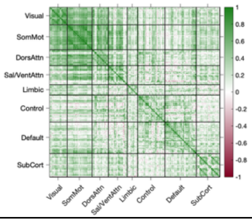	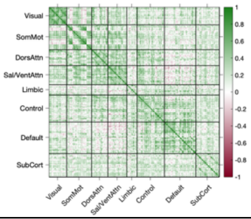	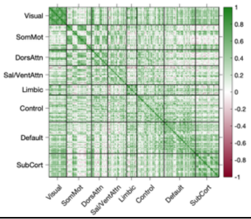

Age 26-27	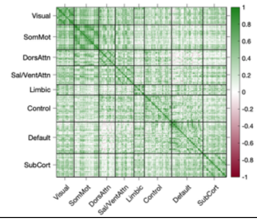	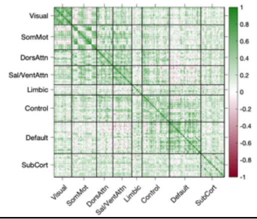	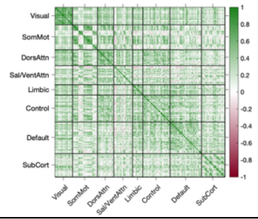
Age 27-28	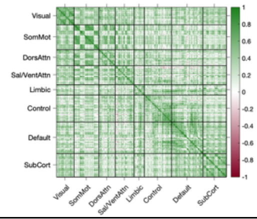		

Table S4: Overview of FC matrices along all groups and across development. Rows: left: Healthy Controls, middle: PPS(-), right: PPS(+); Columns: Window Centers at age 12.5 - 27.5

Overview of all ICC matrices for each window

	Controls	PPS(-)	PPS(+)
Age 12.5	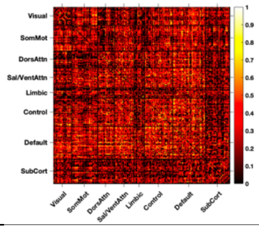	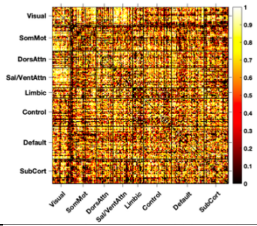	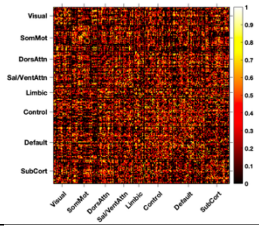
Age 13.5	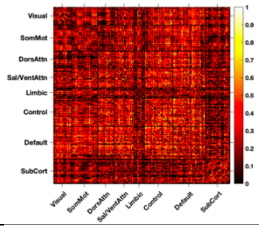	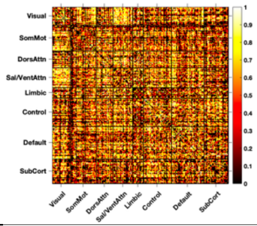	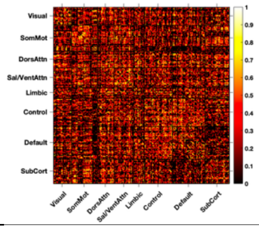
Age 14.5	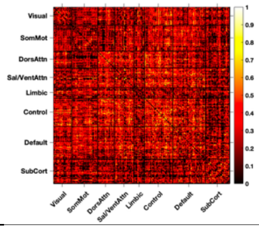	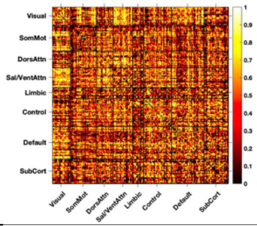	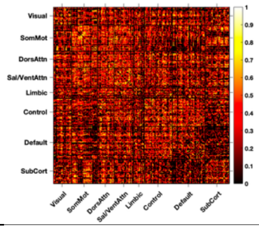
Age 15.5	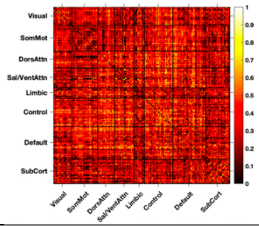	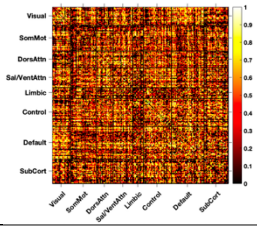	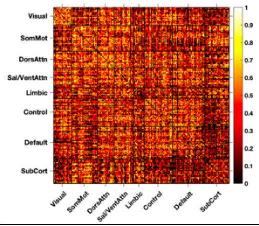

Age 16.5

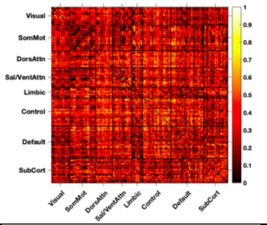

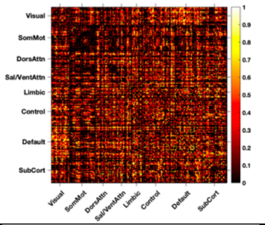

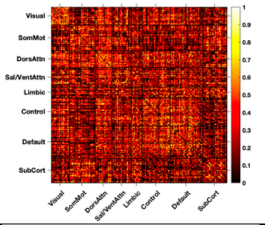

Age 17.5

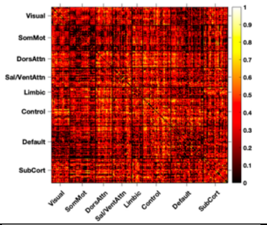

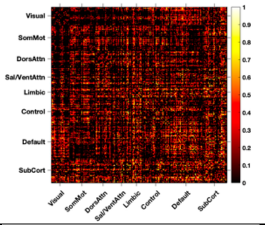

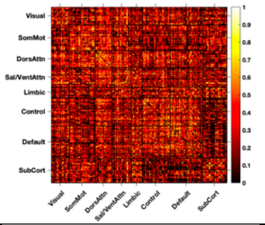

Age 18.5

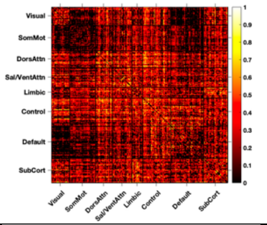

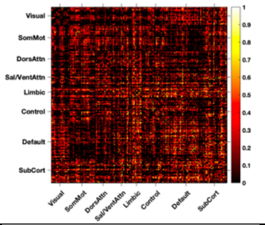

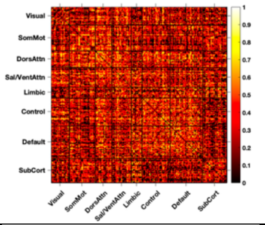

Age 19.5

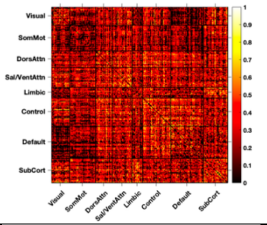

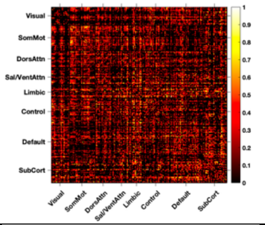

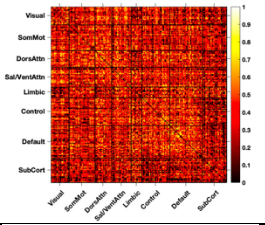

Age 20.5

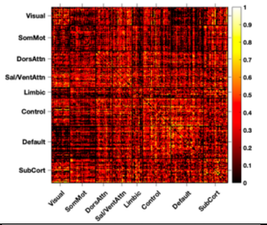

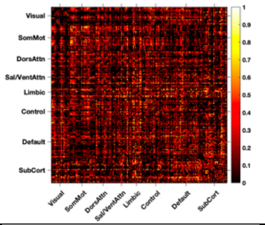

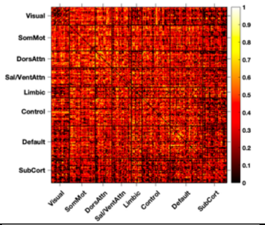

Age 21.5

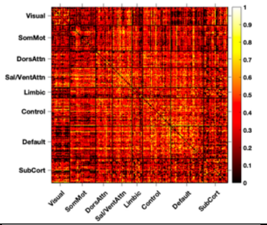

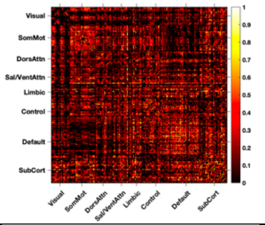

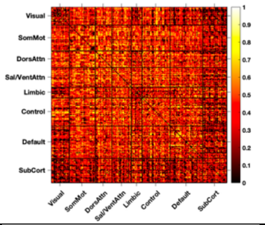

Age 22.5

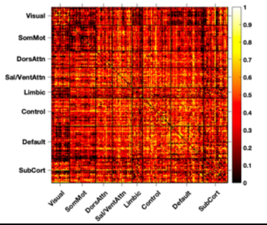

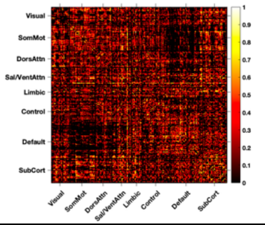

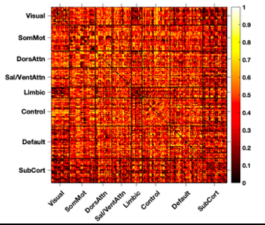

<table>
<tr><td>Age 23.5</td><td>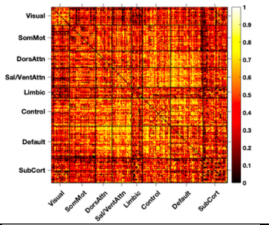</td><td>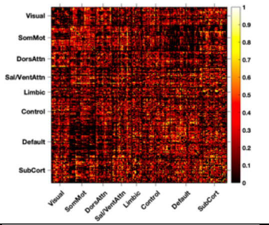</td><td>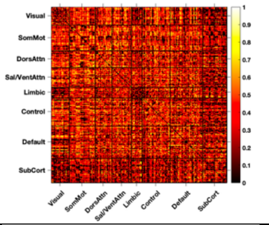</td></tr>
<tr><td>Age 24.5</td><td>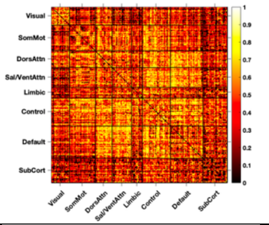</td><td>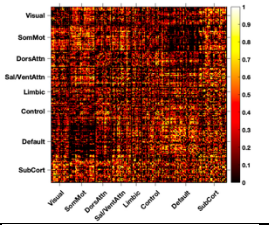</td><td>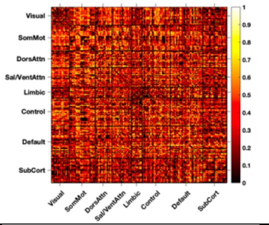</td></tr>
<tr><td>Age 25.5</td><td>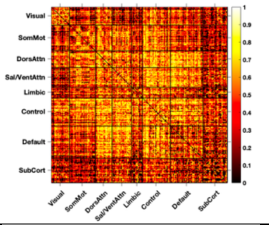</td><td>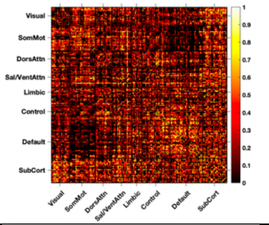</td><td>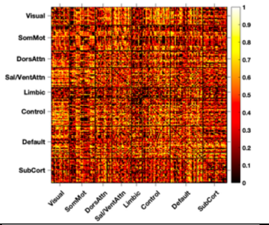</td></tr>
<tr><td>Age 26.5</td><td>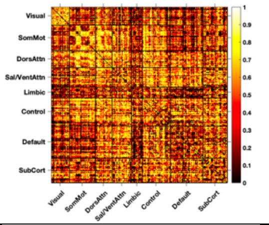</td><td>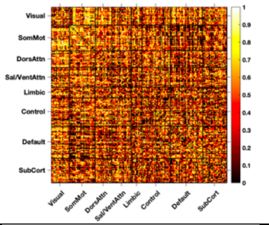</td><td>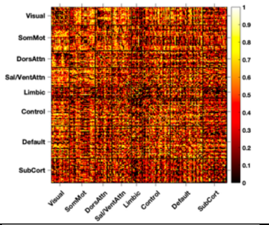</td></tr>
<tr><td>Age 27.5</td><td>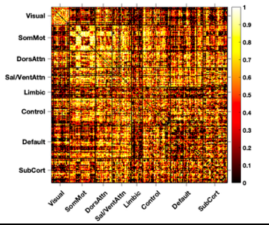</td><td>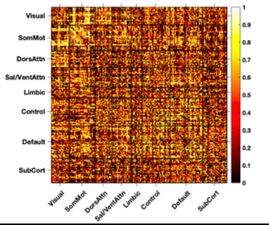</td><td>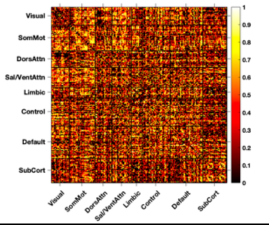</td></tr>
</table>

Table S5: Overview of ICC matrices along all groups and across development. Rows: left: Healthy Controls, middle: PPS(-), right: PPS(+); Columns: Window Centers at age 12.5 - 27.5

Assessment of motion-related stability

To evaluate whether head motion could account for the observed group differences in functional stability, we applied the same longitudinal ICC framework to mean framewise displacement (FD). ICC values were computed across consecutive visit pairs within each age window and group. This analysis assesses the temporal stability of motion itself.

We note that in controls, the developmental trajectory of FD-ICC partially resembles that of FC-ICC, suggesting that global measures of functional stability may be influenced to some extent by general data quality or motion-related factors. However, this relationship is not consistently observed across groups, as PPS(–) and PPS(+) show divergent FD-ICC and FC-ICC trajectories.

While global ICC measuresa may partially reflect motion-related variability, particularly in controls, the dissociation between FD-ICC and FC-ICC trajecto§ries in clinical groups, together with the presence of structured multivariate patterns in PCA space, argues against motion as the primary driver of the reported effects.

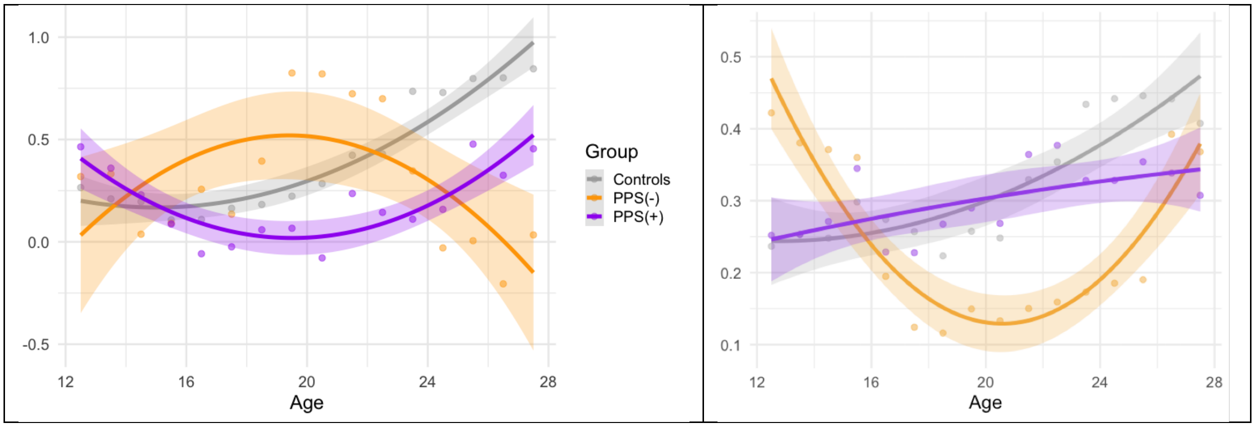

*Figure S9: Comparison ICC analysis in PPS(-), PPS(+) and controls: **Left**: ICC results of FD (framewise displacement) measure. **Right**: ICC results of FCs (Functional Connectomes).*

Overview PCA results

For the FC-ICC combined PCA only window numbers 3-16 were used (e.g. age 14.5-27.5)

PC	FC Loadings	ICC Loadings
PC 1	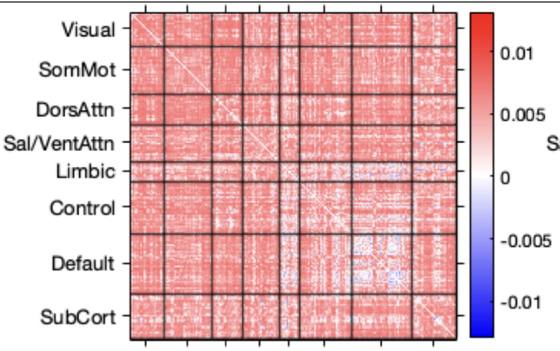	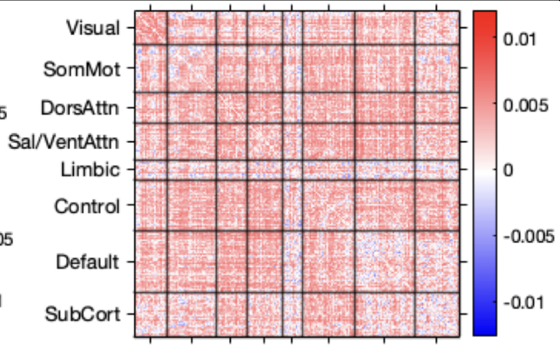
PC 2	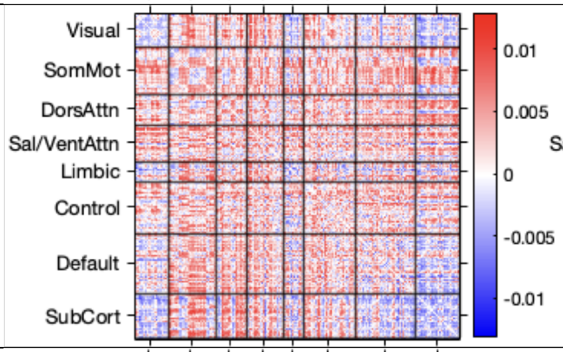	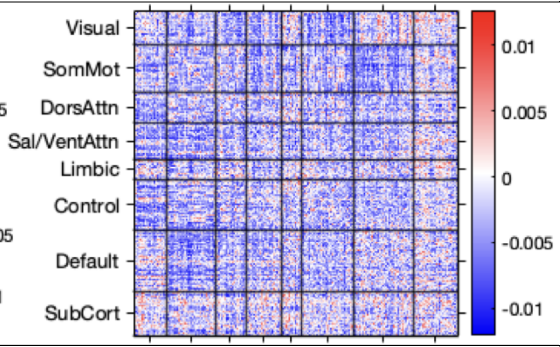
PC 3	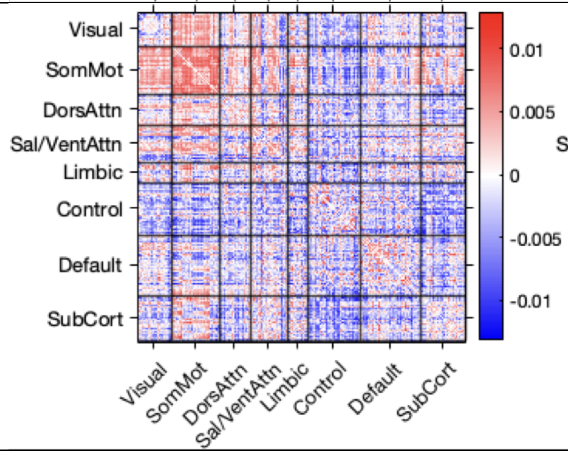	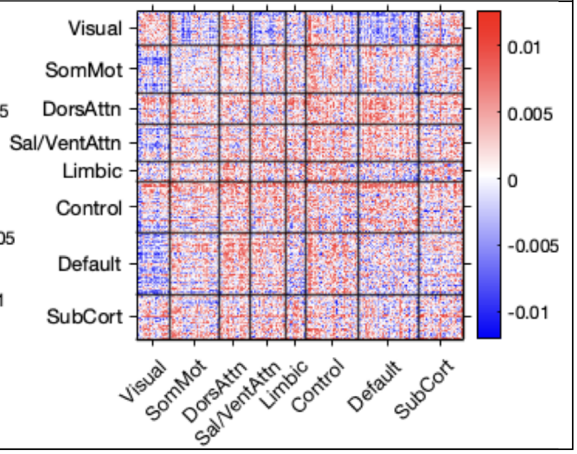

Figure S10: FC loadings (left column) and ICC loadings (right column) of the three principal components

Component	Expl. Var [%]	FC/ICC [%]	Interpretation Label	FC Dominance	ICC Dominance
PC1	27.6	70/30	Sensory-Association Axis (Global hierarchical integration)	Sensory > Association	Midline ICC↑
PC2	10.4	47/53	Emotional–Cognitive Balance (Affective–Sensorimotor Integration)	Motor / cingulate ↑, DMN ↓	Limbic–subcortical ICC↑
PC3	8.9	49.3/50.7	Executive–Sensory Control (Fronto-Striatal – Sensory Integration)	Sensory (auditory) ↑, PFC ↓	Fronto-striatal ICC↑

Table S6: Overview first 3 principal components, columns from left to right: Component, Explained Variance, Contribution of FC and ICC data on this component, Neurophysiological interpretation, Dominant pattern in FC, Dominant pattern in ICC

Assessment of group separation in PCA space

Separation between groups was assessed by computing bootstrap distributions of distances between group-specific PCA representations in the joint FC–ICC space. For each bootstrap iteration, distances were computed between corresponding bootstrap samples from each group and normalized by the 95% confidence radius of a multivariate Gaussian distribution (χ^2 with 3 degrees of freedom). This yielded a separation ratio, where values greater than 1 indicate that the distance between groups exceeds the expected 95% confidence extent. Group separation was considered robust when the lower bound of the 95% confidence interval exceeded 1.

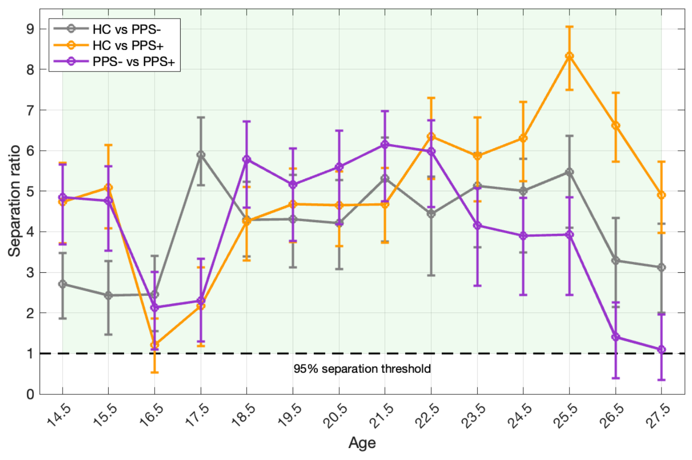

Figure S11: Developmental trajectories of the overlap ratio for each group pair across age. The dashed horizontal line indicates the 95% separation threshold (ratio = 1), and the shaded region highlights separation. Error bars represent 95% confidence intervals estimated from bootstrap resampling.

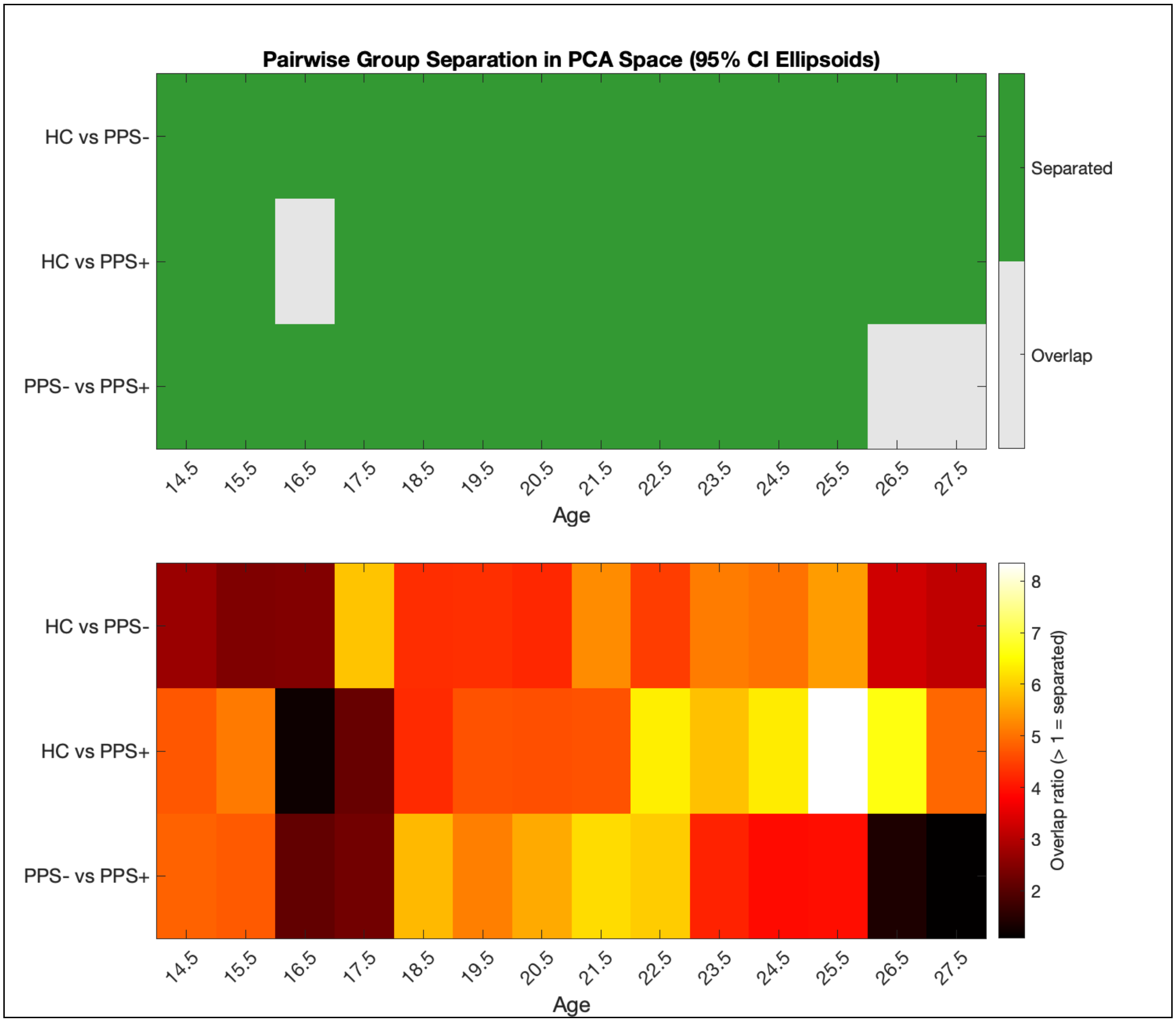

*Figure S12: **Top:** Binary separation map indicating whether pairs of groups (HC vs PPS–, HC vs PPS+, PPS– vs PPS+) are significantly separated at each age window. Separation is defined as non-overlapping 95% confidence ellipsoids derived from bootstrap resampling in the joint FC–ICC PCA space. **Bottom:** Continuous overlap ratio between group centroids, computed as the Mahalanobis distance normalized by the 95% confidence boundary. Values > 1 indicate separation, whereas values < 1 indicate overlap.*

Bootstrap ratio maps of FC and ICC loadings

To assess the stability of edge contributions to each principal component, we computed bootstrap ratios for the reconstructed FC and ICC matrices. Specifically, for each edge, the bootstrap ratio was defined as the ratio of the bootstrap mean to its standard error across resamples (n = 1000). This measure reflects the reliability of each connection's contribution to the component, analogous to a z-score. To facilitate interpretation, we additionally applied a commonly used threshold (|bootstrap ratio| > 2.3, approximately corresponding to a 95% confidence interval), retaining only the most stable connections.

PC	FC Loadings	ICC Loadings
PC1	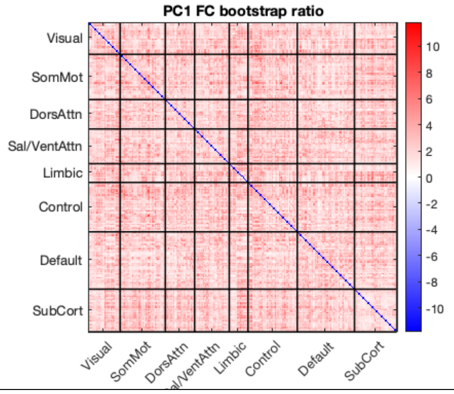	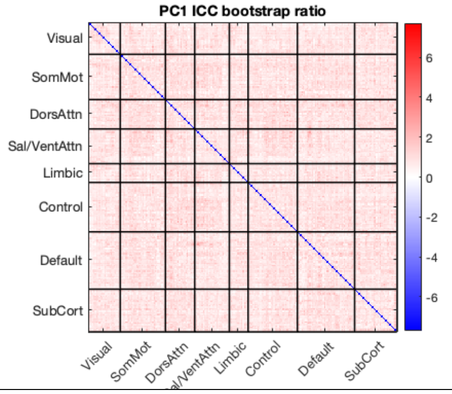
PC2	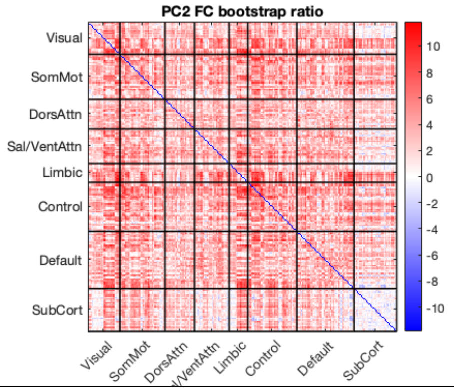	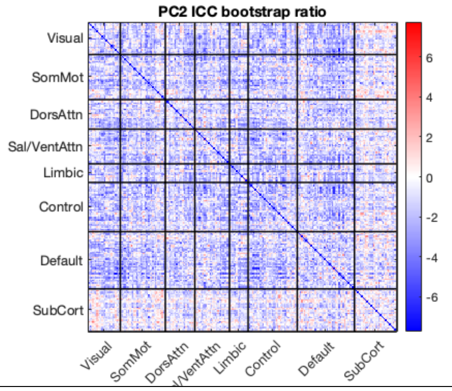
PC3	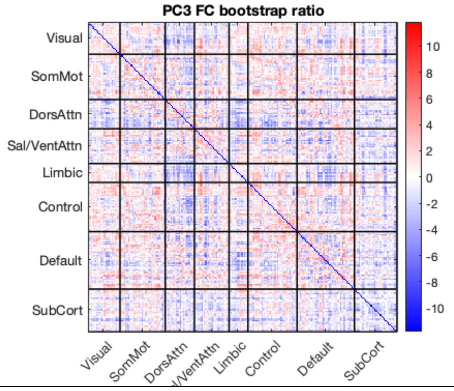	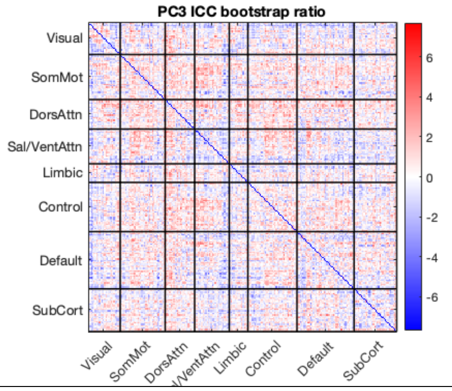

Figure S13: Full bootstrap ratio matrices for FC (left column) and ICC (right column) for the first three principal components. Bootstrap ratios were computed as the ratio of the bootstrap mean to its standard error, reflecting the stability of each edge contribution.

PC	FC Loadings	ICC Loadings
PC1	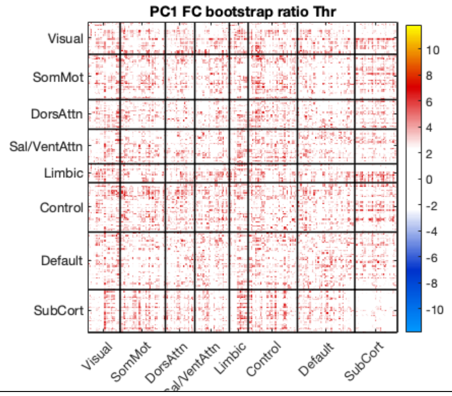	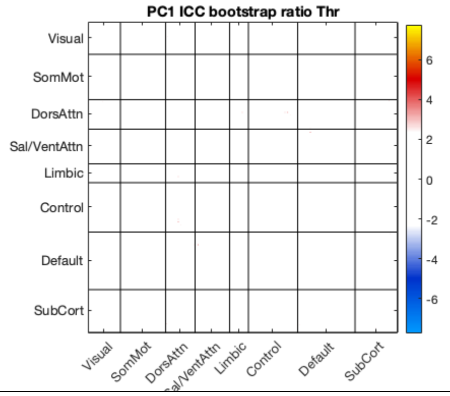
PC2	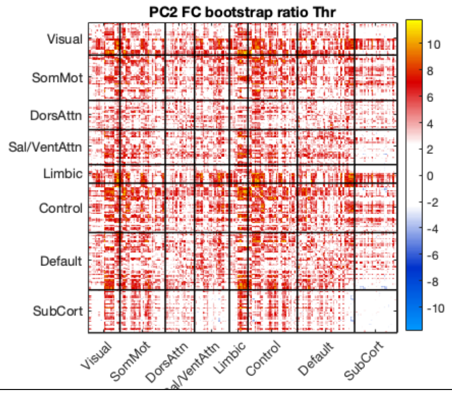	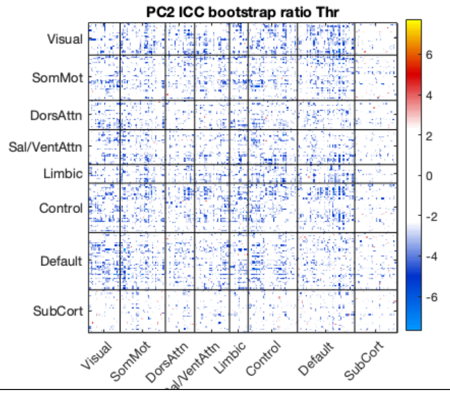
PC3	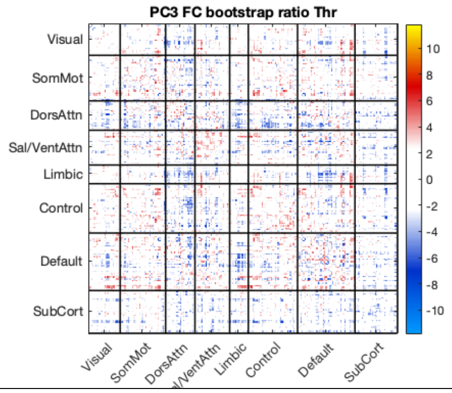	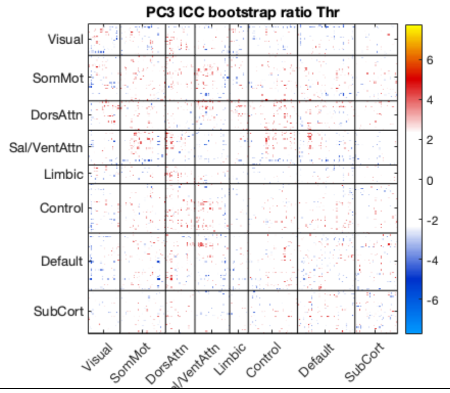

Figure S14: Thresholded bootstrap ratio matrices, where only edges exceeding the stability threshold (|bootstrap ratio| > 2.3) are retained, and all other values are set to zero. These maps highlight the most reliable connections contributing to each principal component.

[1] M. Schaer *et al.*, “Deviant trajectories of cortical maturation in 22q11.2 deletion syndrome (22q11DS): a cross-sectional and longitudinal study.,” *Schizophrenia Research*, vol. 115, no. 2, pp. 182–190, Dec. 2009, doi: 10.1016/j.schres.2009.09.016.
[2] V. Mancini, D. Zöller, M. Schneider, M. Schaer, and S. Eliez, “Abnormal Development and Dysconnectivity of Distinct Thalamic Nuclei in Patients With 22q11.2 Deletion Syndrome Experiencing Auditory Hallucinations,” *Biological Psychiatry: Cognitive Neuroscience and Neuroimaging*, 2020, doi: 10.1016/j.bpsc.2020.04.015.
[3] F. Delavari *et al.*, “Dysmaturation Observed as Altered Hippocampal Functional Connectivity at Rest Is Associated With the Emergence of Positive Psychotic Symptoms in Patients With 22q11 Deletion Syndrome,” *Biological Psychiatry*, vol. 90, no. 1, pp. 58–68, Jul. 2021, doi: 10.1016/j.biopsych.2020.12.033.
[4] J. Ashburner and K. J. Friston, “Unified segmentation,” *NeuroImage*, 2005, doi: 10.1016/j.neuroimage.2005.02.018.
[5] A. Schaefer *et al.*, “Local-Global Parcellation of the Human Cerebral Cortex from Intrinsic Functional Connectivity MRI,” *Cerebral Cortex*, vol. 28, no. 9, pp. 3095–3114, Sep. 2018, doi: 10.1093/cercor/bhx179.
[6] Y. Tian, D. S. Margulies, M. Breakspear, and A. Zalesky, “Topographic organization of the human subcortex unveiled with functional connectivity gradients,” *Nat Neurosci*, vol. 23, no. 11, pp. 1421–1432, Nov. 2020, doi: 10.1038/s41593-020-00711-6.
[7] J. Ashburner, “A fast diffeomorphic image registration algorithm,” *NeuroImage*, vol. 38, no. 1, pp. 95–113, Oct. 2007, doi: 10.1016/j.neuroimage.2007.07.007.
[8] J. D. Power, K. A. Barnes, A. Z. Snyder, B. L. Schlaggar, and S. E. Petersen, “Spurious but systematic correlations in functional connectivity MRI networks arise from subject motion,” *NeuroImage*, 2012, doi: 10.1016/j.neuroimage.2011.10.018.
